# Necroptosis-related LncRNAs in skin cutaneous melanoma: evaluating prognosis, predicting immunity, and guiding therapy

**DOI:** 10.1186/s12885-023-11246-x

**Published:** 2023-08-14

**Authors:** Jianlan Liu, Binlin Luo, Pengpeng Zhang, Keyu Jiang, Zuoqiong Hou, Xiaojian Cao, Jian Tang

**Affiliations:** 1 Department of Orthopedics, The First Affiliated Hospital of Nanjing Medical University, Nanjing, China; 2 Department of Plastic and Burns Surgery, The First Affiliated Hospital of Nanjing Medical University, Nanjing, China; 3 Department of Thoracic Surgery, The First Affiliated Hospital of Nanjing Medical University, Nanjing, China

**Keywords:** Melanoma, Necroptosis, Long non-coding RNA, Immunotherapy, Prognosis

## Abstract

**Background:**

An increasing amount of research has speculated that necroptosis could be a therapeutic strategy for treating cancer. However, understanding the prognostic value of the necroptosis-related long non-coding RNAs (NRLs) in skin cutaneous melanoma (SKCM, hereafter referred to as melanoma) remains poor and needs to be developed. Our research aims to construct a model based on NRLs for the prognosis of patients with melanoma.

**Methods:**

We obtained the RNA-seq and clinical data from The Cancer Genome Atlas (TCGA) database and retrieved 86 necroptosis-related genes from the GeneCards database. The lncRNAs associated with necroptosis were identified via the Pearson correlation coefficient, and the prognostic model of melanoma was constructed using LASSO regression. Next, we employed multiple approaches to verify the accuracy of the model. Melanoma patients were categorized into two groups (high-risk and low-risk) according to the results of LASSO regression. The relationships between the risk score and survival status, clinicopathological correlation, functional enrichment, immune infiltration, somatic mutation, and drug sensitivity were further investigated. Finally, the functions of AL162457.2 on melanoma proliferation, invasion, and migration were validated by in vitro experiments.

**Results:**

The prognostic model consists of seven NRLs (EBLN3P, AC093010.2, LINC01871, IRF2-DT, AL162457.2, AC242842.1, HLA-DQB1-AS1) and shows high diagnostic efficiency. Overall survival in the high-risk group was significantly lower than in the low-risk group, and risk scores could be used to predict melanoma survival outcomes independently. Significant differences were evident between risk groups regarding the expression of immune checkpoint genes, immune infiltration, immunotherapeutic response and drug sensitivity analysis. A series of functional cell assays indicated that silencing AL162457.2 significantly inhibited cell proliferation, invasion, and migration in A375 cells.

**Conclusion:**

Our prognostic model can independently predict the survival of melanoma patients while providing a basis for the subsequent investigation of necroptosis in melanoma and a new perspective on the clinical diagnosis and treatment of melanoma.

**Supplementary Information:**

The online version contains supplementary material available at 10.1186/s12885-023-11246-x.

## Introduction

Skin cutaneous melanoma (SKCM, hereafter referred to as melanoma) is a devastating form of skin cancer whose morbidity is significantly increasing worldwide. According to statistics, there were 324,635 new cases and 57,043 cancer deaths worldwide in 2020 [1]. Advances in immunotherapy and new targeted agents have revolutionized treatment modalities for melanoma patients [2]. However, the accompanying drug resistance and adverse drug reactions still prevent many patients from benefiting from these novel treatment options [2]. Therefore, locating novel biomarkers and drug targets is imperative to optimize treatments and improve the prognosis of melanoma patients.

As early as 2005, Degterev first proposed necroptosis, a specialized form of programmed cell death that, unlike apoptosis or necrosis, is triggered by blocking apoptosis [3, 4]. Necroptosis is a form of lytic cell death that does not require caspase activity; it occurs when the death receptor tumor necrosis factors bind to their death receptor ligands, inhibiting or blocking the apoptotic pathway [3]. Receptor-interacting protein kinase1 (RIPK1), RIPK3, and mixed lineage kinase domain-like protein (MLKL) are sequentially activated in the necroptosis pathway [5]. Thus far, the tumor necrosis factor α (TNFα) / tumor necrosis factor receptor (TNFR) signaling pathway has been investigated intensively. RIPK1 is a signal molecule essential for activating RIPK3 in the TNF-induced necroptosis pathway [3]. RIPK3 acts as an integrative signal for necroptosis requirements. It can interact with other signal molecules and participate in different necroptosis pathways [3]. MLKL is considered a pseudokinase because it lacks two of the three conserved catalytic residues on its kinase-like domain [3]. The whole process culminates in the disruption of cell membrane integrity, organelle swelling, and leakage of contents [5]. Given the available information, we know that the regulation of necroptosis is crucial for tumorigenesis, metastasis, infection, and inflammatory reactions [3, 5, 6]. Expression of RIPK3 is extremely low in melanoma development, but plays a permissive role inhibiting apoptosis proteins (IAP) antagonist-induced necroptosis in malignant melanoma. Reconstituting RIPK3 can reactivate the RIPK1/RIPK3/MLKL signaling pathway, which can eventually overcome the resistance of melanoma to necroptosis [7]. However, few researchers have investigated the association between necroptosis and melanoma patient prognosis.

Long non-coding RNA (lncRNA) is a transcript greater than 200 nucleotides in length. There are approximately 18,000 lncRNAs in the human genome, yet less than 4% have specific functions [8]. Numerous characteristic lncRNAs exhibit regulatory effects in several biological processes, including gene expression regulation, alternative splicing, and chromatin remodelling. There is mounting evidence implicating lncRNA in inflammatory signaling pathways and diseases [9]. Furthermore, lncRNA has a notable impact on the processes of malignancies, including their genesis, invasion, growth, metastasis, diagnosis, and treatment [10]. In this context, lncRNA has been recognized as a potential biomarker for cancers in bodily fluids. Non-apoptotic cell death signaling mechanisms in melanoma have been the subject of growing research interest.

Nevertheless, to our knowledge, there is a paucity of research exploring the link between necroptosis-related lncRNAs (NRLs) and survival in melanoma patients. Therefore, we conducted in-depth research and employed bioinformatics to investigate the association between NRLs and melanoma. The study aims to gain insight into the application of these lncRNAs in terms of predictive value, immunological infiltration, functional enrichment, tumor mutational burden (TMB), immunotherapy and drug sensitivity. Ultimately, we inhibited the expression of lncRNA AL162457.2 in vitro to determine its effect on melanoma cell proliferation and migration. Our findings introduce a fresh perspective on the impact of necroptosis on melanoma and suggest that NRLs can be exploited for the diagnosing and predicting the prognosis of this condition.

## Materials and methods

### Extraction of original clinical data

The TCGA database was essential for obtaining data matrices of melanoma RNA sequence datasets (HTSeq-FPKM) and the relevant clinical data. The following are the requirements for inclusion: (1) patients had a definite diagnosis of melanoma; (2) patients possessed complete lncRNA and clinical information. Altogether, the clinical information and lncRNA data of 450 individuals, who satisfied the selection criteria, were downloaded for further research.

### Identification of LncRNAs associated with necroptosis

We searched the GeneCards database with the keyword “necroptosis” and extracted necroptosis-related gene (NRG) lists with a relevance score ≥ 1. We obtained the profiles of 86 NRGs and subsequently performed the co-expression analysis of lncRNAs and NRGs in melanoma with Strawberry Perl and the “limma” R package. The interaction between the expression levels of the 86 NRGs and those of lncRNAs was determined by performing a Pearson correlation analysis. The criteria of correlation coefficients with absolute values > 0.4 and P-values < 0.001 (|R| > 0.4, P < 0.001) were identified as NRLs [11]. We visualized the co-expression networks between lncRNAs and NRGs using the R package “igraph”.

### Construction of the necroptosis-related LncRNAs prognostic model

Firstly, we implemented a univariate Cox regression analysis to assess the relationship between NRLs and survival status in melanoma patients. Alternative lncRNAs were subsequently incorporated into the Least Absolute Shrinkage and Selection Operator (LASSO) regression model (with the “glmnet” R package) to develop a prognostic model. Next, seven lncRNAs associated with survival were identified for subsequent analysis. The median risk score was applied to stratify individuals into high- and low-risk subgroups. The following algorithm was adopted to generate risk scores: risk score = ∑^n^_*i*=1_β_*i*_ ∗ (expression of lncRNA_*i*_). The disparity in survival rates between the two risk groups was illustrated by the Kaplan–Meier (K-M) curves. Meanwhile, the distribution and scatter plots of patient risk scores were used to depict the intricate relationships between survival status and risk score.

### Assessing the independent prognostic value of the risk model

The t-distributed Stochastic Neighbor Embedding (t-SNE) algorithm and principal component analysis (PCA) were employed to display the stratification of patients in distinct risk categories according to NRLs expression. We applied univariate and multivariate Cox regression analysis to determine the influence of risk score and clinicopathological characteristics (Age, Gender, Stage, T, M and N) on the overall survival (OS). We aimed to ascertain whether the risk score significantly predicted the outcomes. The predictive accuracy and efficiency of the established prognostic model were validated by the calibration curve and the receiver operating characteristic (ROC) curve. Using the “survival” and “time ROC” software packages, 1 to 5 years ROC curves were plotted for the model. To verify the universality, we conducted a stratified analysis to further evaluate the risk model’s predictive capacity in each clinical subgroup. A nomogram incorporating calibration plots was utilized (using the “rms” R package) to forecast the OS for melanoma patients to demonstrate whether the prediction outcome was consistent with the actual survival status of patients.

### Tumor classification using prognostic necroptosis-related LncRNAs

Utilizing the expression patterns of prognostic NRLs as a reference, we employed the K-means clustering method to stratify melanoma patients into separate subtypes to achieve the highest intra-group correlation and the lowest intergroup correlation. The gene expression profiles and clinical characteristics were visualized with a heatmap. The overall survival curve was then plotted from the different clusters, and the Sankey diagram was employed to illustrate the connection between clusters and risk scores.

### Differential analysis of immune landscape and tumor mutational burden in different groups

Firstly, the CIBERSORT algorithm was employed to evaluate the immune infiltration of individual melanoma patients, with boxplots presenting the associations between the risk model and immunocyte infiltration [12]. Next, we conducted a single-sample gene set enrichment analysis (ssGSEA) to calculate the infiltration scores of 16 immune cells and assess the activity of 13 immune-related functions between the two risk groups. The ESTIMATE algorithm was applied to infer the proportion of stromal and immune cells in each melanoma sample, providing insight into tumor-immune interactions [13]. We obtained 47 immune checkpoint-associated genes from previously published literature. The variation between immune-related genes in the two risk populations was analyzed using the rank-sum test, and box plots were used to plot genes showing distinct dissimilarities between the two groups. Melanoma mutation information (TCGA.SKCM.varscan.6c961926-7792-42fa-9a16-c62f60e2557b.DR-10.0.somatic) were retrieved from the TCGA database, and somatic mutations were assessed utilizing Mutation Annotation Format (MAF) tools [14]. We acquired the TMB of each melanoma patient based on somatic mutations. Spearman correlation analysis was employed to analyze the association between TMB and risk scores, and the survival probability of patients stratified by risk score and TMB were compared. In addition, the Gene Set Variation Analysis (GSVA) [15] and gene set enrichment analysis (GSEA) were conducted, to identify pathways with substantial correlation in both groups and visualized five representative pathways involved in necroptosis in the Kyoto Encyclopedia of Genes and Genomes (KEGG) [16, 17].

### Immunotherapy response and drug sensitivity evaluation across various risk groups

Inhibitory receptors (IRs) such as programmed cell death protein 1 (PD-1), programmed cell death-Ligand 1 (PD-L1) and cytotoxic T lymphocyte-associated protein 4 (CTLA4) have progressed to primary targets for cancer immunotherapy, which have been documented in multiple major clinical trials [18]. Extensive pre-clinical evidence and mechanistic studies have prompted lymphocyte-activation-gene-3 (LAG3) as the third checkpoint to be addressed in the clinic, with almost a dozen therapies under investigation [19]. Hence, the differences in PD-1/PD-L1, CTLA4 and LAG3 expression levels across the varying risk groups were analyzed using the Wilcoxon test. Immunophenoscore (IPS), positively correlated with tumor immunogenicity, and is considered a better predictor of immune checkpoint inhibitors (ICIs) response [20]. IPS includes four major components: effector cells, immunosuppressive cells, major histocompatibility complexes (MHC) molecules and immunomodulators. The Cancer Immunome Atlas (TCIA, https://tcia.at/) is essential for obtaining IPS in melanoma patients. Half-maximal inhibitory concentrations (IC_50_) of frequently used chemotherapeutics in the two risk groups were determined using the “pRRophetic” R package. Disparities were calculated statistically, and scatterplots were created from the obtained correlations. The purpose of this was to screen for potentially effective drugs for melanoma treatment and evaluate the effectiveness of this model, in terms of its clinical application for melanoma patients.

### Patients and samples

With the patient’s informed consent, six melanoma tissue specimens and adjacent normal tissues were surgically obtained at the Department of Plastic and Burns Surgery, First Affiliated Hospital of Nanjing Medical University. None of the patients received any treatment before surgery. All study procedures were carefully adhered to the Helsinki declaration for the use of human participants and were authorized by the Ethics Committee of the hospital (No.2022-SR-465).

### Cell culture and transfection

We obtained human keratinocyte cells (HaCaT) together with human malignant melanoma cell line A375, from The Cell Bank of Type Culture Collection of the Chinese Academy of Science, and cultured them in DMEM (Gibco BRL, Rockville, MD, USA) + 10% fetal bovine serum (FBS, Gibco BRL, Rockville, MD, USA) + 1% penicillin-streptomycin solution at 37°C with 5% CO_2_. Ribobio (Guangzhou, China) was commissioned to synthesize small interfering RNA (siRNA) and its negative control (NC). The target sequences of siRNA for A162457.2 were 5’ – ACCAGCAAACACCTACAAT (si-A162457.2-1) and 5’ –TCCAATGGATTCCCAGAAA (si-A162457.2-2). A375 cells were treated with si-A162457.2 or siNC using Lipofectamine 3000 following the protocol provided by the manufacturer (Invitrogen; Carlsbad, CA, USA).

### RNA extraction and quantitative real-time PCR (qRT-PCR)

The total RNA was isolated from cells or tissues by TRIzol (15,596,018, Thermo, Waltham, MA, USA). The quantity and quality of RNA were determined spectrophotometrically and subsequently reversed transcription into cDNA following the PrimeScript™ RT reagent kit (R232-01, Vazyme, Nanjing, China). The expression of AL162457.2 was determined by SYBR Green Master Mix (Q111-02, Vazyme, Nanjing, China). qRT-PCR was then conducted on the LightCycler 480 Real-Time PCR System (Roche Diagnostics, Basel, Switzerland), with GAPDH serving as the internal control for AL162457.2. To calculate the level of AL162457.2, the 2^−∆∆CT^ method was used. All primers used for qRT-qPCR were produced by Tsingke Biotech (Tsingke, Beijing, China), and the following is a list of primer sequences: AL162457.2 (Forward): TACAAATCAGGAGGAAAA; AL162457.2 (Reverse): AGTGGAGAGATGAGGGTG. GAPDH (Forward): GGCCTCCAAGGAGTAAGACC; GAPDH (Reverse): AGGGGAGATTCAGTGTGGTG.

### Cell proliferation assay and EdU assay

We utilized the Cell Counting Kit-8 (CCK-8) assay to explore A375 cell proliferation. The transfected cells were planted at a density of 2000 cells/well in a 96-well plate overnight (at 37 °C with 5% CO_2_). The cells were incubated with 10 µL of CCK-8 labelling reagent (A311-01, vazyme, Nanjing, China) and 90 µL of serum-free medium per well in darkness at 37 °C for 2 h. Absorbance was determined at 450 nm wavelength with the enzyme-labelled meter (A33978, Thermo, Waltham, MA, USA). After that, A375 cells were stained with the EdU assay kit (Ribobio, Guangzhou, China) following the manufacturer’s directions. three randomly selected fields were photographed by a fluorescence microscope. Lastly, the EdU-positive cells were counted and quantified through the ImageJ software. Concerning the colony formation experiment, about 500 transfected A375 cells were seeded into each well of the 6-well plate and given time to form into colonies. Next, cells were washed with PBS and fixed with 4% paraformaldehyde for 15 min. Staining crystalline violet for 20 min, allowing it to dry at room temperature, and then counting the number of cells under an inverted microscope yielded the desired results.

### Transwell migration and wound healing assays

In a 24-well transwell plate, A375 cells were resuspended in 200 µL medium without FBS and inserted into the upper chamber, while 600 µL of medium containing 10% FBS was placed in the lower chamber. Invasion and migration assays were conducted with or without Matrigel coating on the transwell chamber (2 mg/ml, BD Biosciences, Franklin Lakes, NJ, USA). Non-invasive cells were eliminated from the top of the membrane after 48 h, whereas invasive cells were fixed with 4% paraformaldehyde and stained with 0.1% crystal violet (Solarbio, Beijing, China). Three different fields of cells were photographed and counted at 200x magnification using a light microscope. Transfected A375 cells were cultured in 6-well plates containing DMEM-free of FBS, and linear wounds were scratched with a 200 µL pipette tip for the wound healing assay. After scratching, cell migration was assessed and photographed at fixed positions at 0 and 48 h under an inverted microscope.

### AL162457.2 target gene prediction

It has been noted that lncRNAs could regulate the transcription of their neighboring protein-coding genes (cis-acting lncRNAs). The mRNAs showed correlated expression patterns with differently expressed lncRNAs and were more likely to be modulated by the lncRNAs (trans-acting lncRNAs) [21]. Therefore, we applied the Pearson correlation analysis to identify co-expressed lncRNAs and mRNA in each comparison group. The lncRNA-mRNA pairs with Pearson correlation score > 0.4 were considered co-expression relationships.

### Statistical analysis

Each experiment was conducted in triplicate. Results were reported as mean ± standard deviation (SD). GraphPad Prism 8.0 software (La Jolla, CA, USA) and the R package (V.4.1.2) were employed for the statistical analysis of the experimental data. The unpaired t-test or one-way ANOVA was implemented to examine the differences between the groups, while the Chi-square (χ^2^) test was intended to evaluate the categorical variables. P < 0.05 was deemed a statistical significance.

## Results

### Necroptosis-related LncRNAs in melanoma patients

**Fig. 1:**
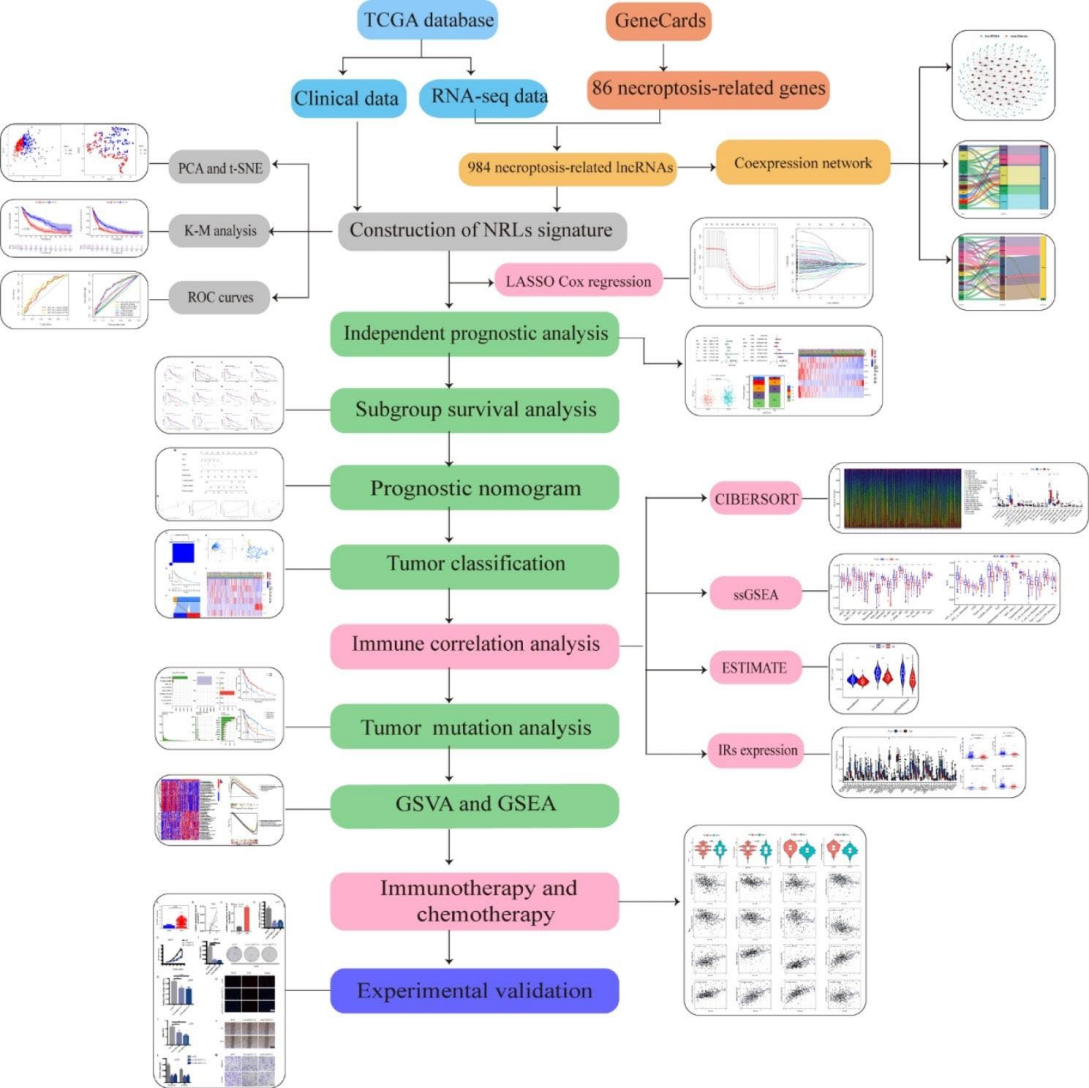
The flowchart of our research

**Fig. 2:**
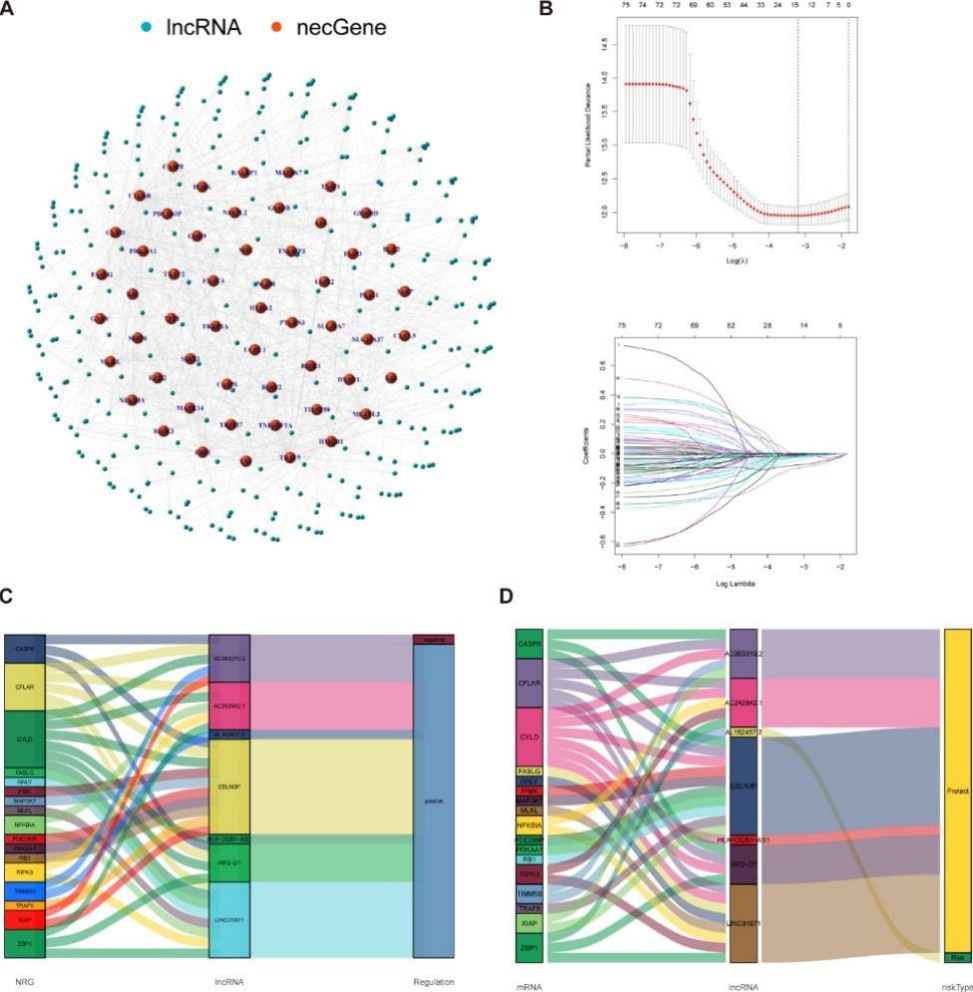
Identification of necroptosis-related lncRNAs in patients with melanoma. (**A**) The co-expression network between necroptosis-related genes and lncRNAs (correlation coefficients > 0.4 and p < 0.001). Orange nodes represent necroptosis-related genes, and the sky blue nodes represent their associated lncRNAs. (**B**) Cross-validation for tuning the parameter in the LASSO regression, the vertical dashed lines indicate are at the optimal log (lambda) value. (**C**) The Sankey diagrams were generated to visualize association networks between prognostic lncRNAs, necroptosis-related genes and regulate status (positive/negative). (**D**)The correlation network of prognostic necroptosis-related lncRNAs and associated mRNAs (risk / protect)

The flowchart of our research was presented in Fig. 1. 14,056 lncRNAs were identified in the TCGA-SKCM database, and a complete list containing 86 NRGs with a relevance score of ≥ 1 was retrieved from the GeneCards database. We ultimately obtained 984 lncRNAs associated with necroptosis (coefficients |R| >0.4 and P < 0.001). The network diagram provided interactive information among necroptosis-associated genes and lncRNAs (Fig. 2A). Univariate Cox regression analysis was conducted for preliminary filtration of the NRLs. Among these, 76 lncRNAs were found to correlate with melanoma OS, and then these lncRNAs were included in the LASSO Cox regression model to further select and validate the best prognostic lncRNAs (Fig. 2B). We found seven NRLs were significantly relevant to melanoma prognosis, and these data served as the foundation for our prognostic model (Table 1). Sankey plots were generated to visualize association networks between the lncRNAs, necroptosis-related genes, and regulation status (Fig. 2C). The network of the prognostic lncRNAs and their associated mRNAs is displayed in Fig. 2D. We can see that necroptosis-related genes negatively regulate only AC093010.2. Meanwhile, only AL162457.2 could function as a risky lncRNA compared to other alternative lncRNAs that may have protective effects.

**Table 1:** Multivariate Cox results of lncRNAs based on TCGA-SKCM data

lncRNA	Coefficient	HR
EBLN3P	-0.03455	0.966038
AC093010.2	-0.02627	0.974076
LINC01871	-0.03997	0.960821
IRF2-DT	-0.15353	0.857677
AL162457.2	0.006489	1.00651
AC242842.1	-0.13438	0.874255
HLA-DQB1-AS1	-0.0755	0.927279

### Establishment and evaluation of a necroptosis-related signature for melanoma prognosis prediction

**Fig. 3:**
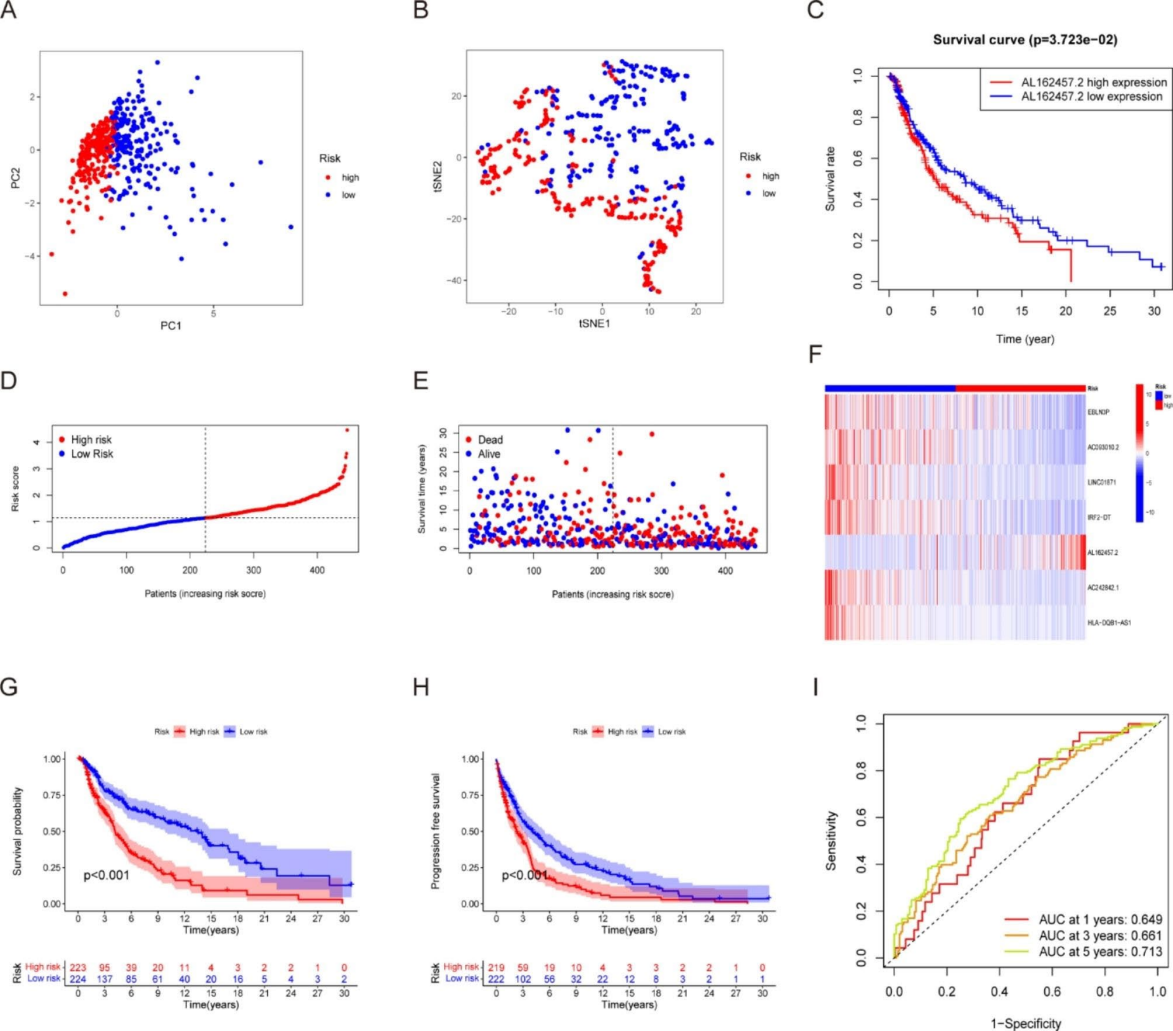
Evaluation of a necroptosis-related signature for melanoma prognosis prediction. (**A**) Principal component analysis (PCA) of melanoma patients according to the risk score. (**B**) T-distributed Stochastic Neighbor Embedding (t-SNE) of melanoma patients according to the risk score. (**C**) Survival curve of melanoma patients with high and low AL162457.2 expression. The distribution of the risk scores (**D**), overall survival status (**E**), and the expression of necroptosis-related lncRNAs (**F**) among melanoma patients was shown. (low-risk population: on the left side of the dotted line; high-risk population: on the right side of the dotted line; green represents the number of survivors, and red represents the number of deaths. The risk from low to high reveals a rising tendency in deaths). Kaplan-Meier overall survival (**G**) and progression-free survival (**H**) curves of the risk stratification groups and the shaded area represent the 95% confidence interval (CI). The clinical outcome in the high-risk group was inferior to those in the low-risk group. (**I**) The area under the curve (AUC) value of the time-dependent ROC curves shows the predictive performance of the risk score, with the 1-year, 3-year, and 5-year AUC being 0.649, 0.661 and 0.713, respectively

Next, the LASSO results were incorporated into a multivariate Cox analysis to produce an individual risk score. The formula used for this analysis was as follows: risk score =(-0.035×EBLN3P) + (-0.026×AC093010.2) + (-0.040×LINC01871) + (-0.154×IRF2-DT) + (0.006×AL162457.2) + (-0.134×AC242842.1) + (-0.076×HLA-DQB1-AS1). We used PCA maps and t-SNE plots to visualize the distribution of patients according to the expression of model lncRNAs (Fig. 3A, B). The results showed that patients with varying risks were placed in distinct categories, suggesting that the constructed model is highly effective in distinguishing between high- and low-risk melanoma. Among the seven lncRNAs, only AL162457.2 overexpression was correlated with a poor prognosis (Fig. 3C). In contrast, melanoma patients with high expression of the other six candidate lncRNAs had a higher chance of survival (Fig. S1). Afterward, we compared the risk score, survival status, survival rate, and relevant expression of lncRNAs amongst the various risk groups (Fig. 3D), showing that high-risk individuals had lower life expectancies (Fig. 3E). A heatmap depicted that AL162457.2 was significantly highly expressed in the high-risk group (Fig. 3F). In K-M survival analyses, patients with low-risk scores fared better than those with high-risk scores with regard to OS and progression-free survival (Fig. 3G, H). The area under the curve (AUC) values for 1-, 3- and 5-year OS were 0.649, 0.661 and 0.713, respectively (Fig. 3I). Based on these findings, our model may play a pivotal role in determining the prognosis of melanoma patients.

### Independent prognostic analysis of the model

**Fig. 4:**
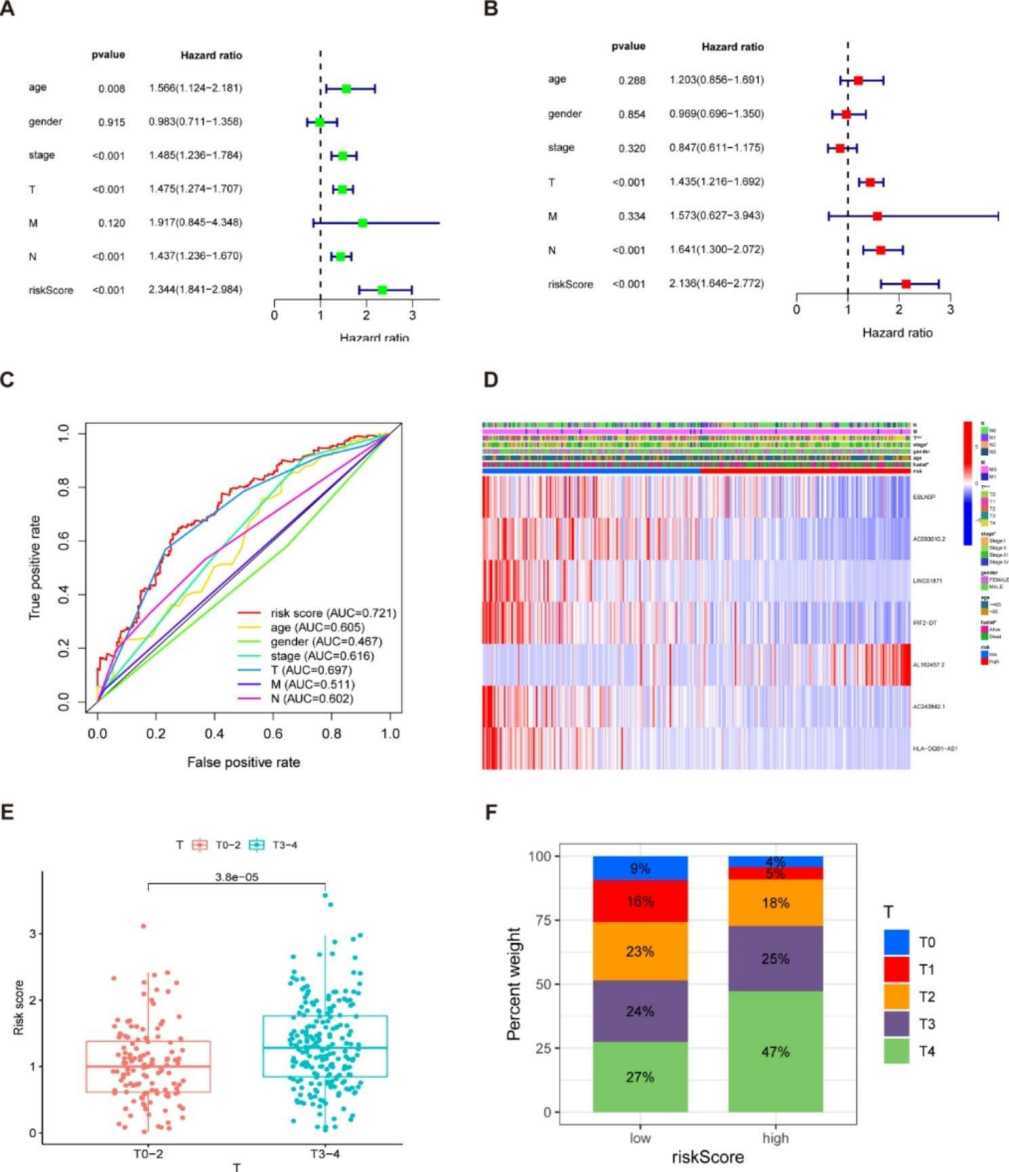
Prognostic values of clinicopathological factors and risk score. (**A**) Univariate Cox regression analysis showed that stage, T, N, and risk score were independent factors for melanoma prognosis. (**B**) Multivariate Cox regression demonstrated that T, N, and risk score were independent prognostic predictors. The risk score is the parameter with the highest HR value in univariate or multivariate Cox analysis. (**C**) The ROC curve of the risk score had the largest AUC of 0.721 compared with the other clinical variables. (**D**) Heatmap (blue: low expression; red: high expression) for the relationship between clinicopathologic characters and the risk groups, indicating that survival status, T, and stage were significantly different between the two groups. (**E**) Correlation analysis between risk score and T status. Patients with melanoma of stage T3-4 had substantially higher risk scores than those with stage T1-2. (**F**) The distribution of T-stage in high- and low-risk populations. Note: *** P ≤ 0.001. ** P ≤ 0.01. * P ≤ 0.05

Univariate and multivariate Cox regression was conducted to determine whether the risk score influences factors in melanoma patients independently of other clinical traits. Table 2 displays the full list of patients’ clinical characteristics that were collected for this investigation. Univariate Cox regression revealed that age, stage, T, N, and risk score had prognostic significance in melanoma patients (Fig. 4A). The multivariate Cox analysis illustrated that, after correcting for other confounding variables, T, N, and risk score were determined to be independent prognostic predictors (Fig. 4B). Furthermore, the risk score is the parameter with the highest hazard ratio (HR) value in univariate or multivariate Cox analysis. For the 5-year ROC of the risk model, the risk score (AUC = 0.721) demonstrated greater prediction accuracy than other conventional clinical and pathological indicators (Fig. 4C), demonstrating the high predictive power of our risk model for melanoma OS. In addition, we generated a heatmap to visualize the clinical features of melanoma patients. Of which, Fig. 4D shows significant differences between the low- and high-risk subgroups in survival status, T status and clinical stage. Patients with stage T3-4 melanoma were related to a significantly higher risk score than those with stage T1-2 (Fig. 4E-F). Moreover, there was a more significant proportion of stage III and IV patients in the high-risk group.

**Table 2:** The clinical characteristics of the patients in the database

Clinical	n	Mean	SD	t	P
Age					
> 65	115	1.318	0.696	2.046151	0.042
≤ 65	221	1.162	0.599		
Gender					
Female	128	1.172	0.626	-0.9746	0.331
Male	208	1.241	0.644		
Stage					
I-II	182	1.223	0.645	0.25043	0.802
III-IV	154	1.206	0.629		
T					
T0-2	128	1.035	0.57	-4.29037	0
T3-4	208	1.326	0.652		
M					
M0	325	1.216	0.636	0.178065	0.862
M1	11	1.178	0.695		
N					
N0	190	1.214	0.643	-0.02312	0.982
N1-3	146	1.216	0.631		

### Subgroup analysis of the prognostic model and construction of prognostic nomogram

**Fig. 5:**
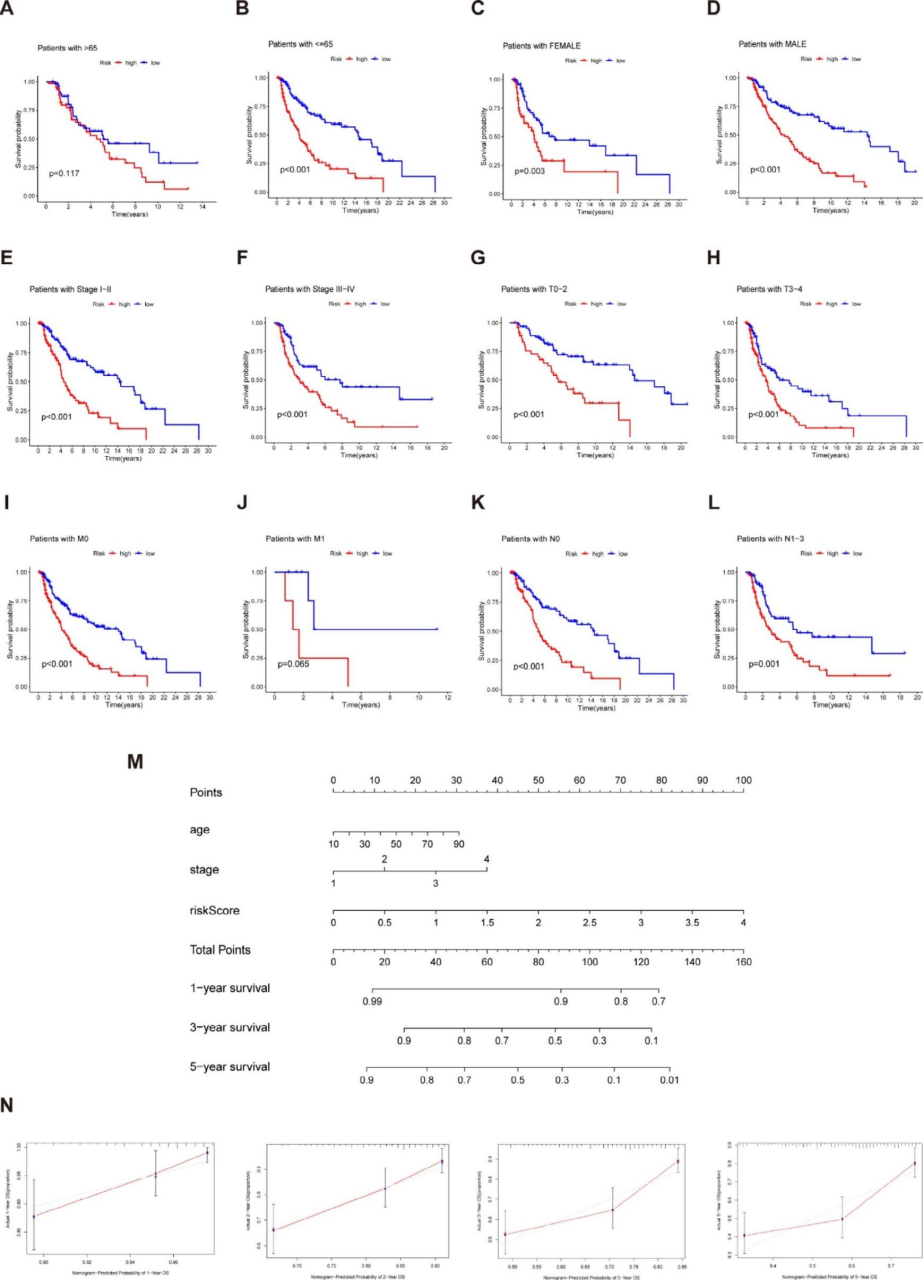
Subgroups analysis of clinical features in the predictive model and construction of nomogram (**A**-**L**) Kaplan–Meier survival plots of high- and low-risk patients in subgroups are based on clinical features. (**M**) A nomogram combing clinicopathological variables and risk score predicted melanoma patients 1, 3, and 5 years OS. (**N**) The calibration plots test consistency between the actual OS rates and the expected survival rates at 1, 2, 3, and 5 years. The closer the solid red line is to the solid grey line, the closer the nomogram prediction probability is to the actual probability

We performed survival analysis for different clinical subgroups to explore whether the established prognostic model could predict OS based on various clinical characteristics. Specific analysis information is as follows: age (≤ 65 or > 65), gender (male or female), clinical stage (stage I-II or stage III-IV), T status (T0-2 or T3-4), M status (M0 or M1), and N status (N0 or N1-3). As depicted in Fig. 5A-L, regardless of age, gender, clinical stage, T status, M status or N status, the OS rates for high-risk patients were consistently lower than those for low-risk individuals. On this basis, we developed a nomogram- a quantitative model for further predicting the incidence of melanoma patients’ 1-, 3- and 5-year OS (Fig. 5M). We also utilized the 1-, 2-, 3- and 5-year calibration plots to demonstrate that the nomogram achieved excellent consistency between actual and predicted risk and could precisely predict the prognosis of melanoma patients (Fig. 5N). Overall, the prognostic model is closely related to melanoma development and may play a pivotal role in patient management.

### Tumor classification based on the necroptosis-related LncRNAs

**Fig. 6:**
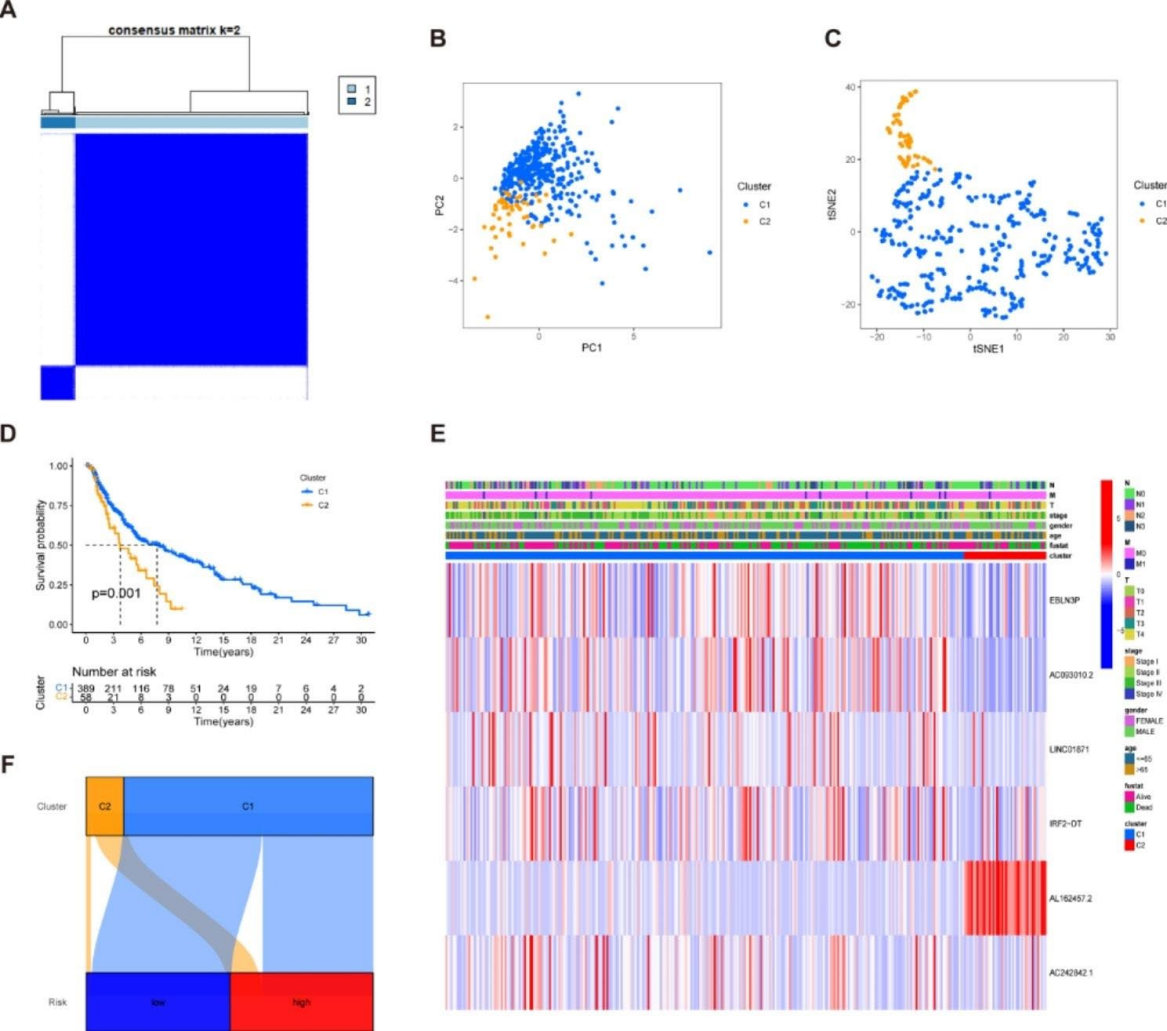
Tumor classification was based on the identified prognostic NRLs. (**A**) 447 melanoma patients were stratified into two clusters according to the consensus clustering matrix (k = 2). (**B**-**C**) Principal component analysis (PCA) and t-distributed Stochastic Neighbor Embedding (t-SNE) algorithm and for clusters. (**D**) Kaplan–Meier curves show C1(blue) and C2 (yellow) survival for melanoma patients. C2 has a worse prognosis than C1 (P = 0.001). (**E**) Heatmap and the clinicopathologic characters of the two clusters. (**F**) Sankey diagram of the relationship between the two clusters and risk groups. Note: *** P ≤ 0.001. ** P ≤ 0.01. * P ≤ 0.05

Next, to investigate the underlying relationships between the expression of the NRLs and melanoma subtypes, we employed the k-means clustering method to categorize melanoma patients into subtypes based on NRLs. By raising the clustering variable (k) from 2 to 10, we obtained the highest intra- and lowest inter-group correlations when k = 2 (Fig. 6A), suggesting that 447 melanoma patients could be successfully separated into two clusters (the detailed classification process can be found in Fig. S2). PCA and t-SNE plots showed that C1 patients could be well isolated from C2 patients (Fig. 6B-C). Comparing the survival rates of patients in the two clusters, we found a significant difference between them, with C1 having a higher survival rate than C2 (Fig. 6D). Furthermore, we generated a heatmap to illustrate the gene expression profile, clinical features, and survival status (Fig. 6E). Sankey diagram indicated that C1 was significantly relevant to the low-risk group, while C2 was implicated in the high-risk group to verify its relationship with risk (Fig. 6F). To investigate the underlying mechanisms of differences in prognosis across subpopulations, we quantified the proportion of different clinicopathological features in the C1 and C2 groups. The percentage bar chart showed that there was no significant difference in the proportion of T, M, and N stages in the two subgroups (Fig. S3). With reference to previous studies, we found that different clusters commonly showed different TME, resulting in various responses to immunotherapy. We conducted immune scores for two clusters, which showed that C1 had higher stromal scores, immune scores, and estimated scores, demonstrating that the TME of C1 was different from C2. As is shown in the heatmap of immune cells in two clusters, C1 was more highly infiltrated by immune cells based on analyses of the different platforms. Additionally, almost all the immune checkpoints had a high expression in C1, such as PDCD1, IDO1 and LAG3 (Fig. S4).

### Immunity and mutation analysis between risk groups

**Fig. 7:**
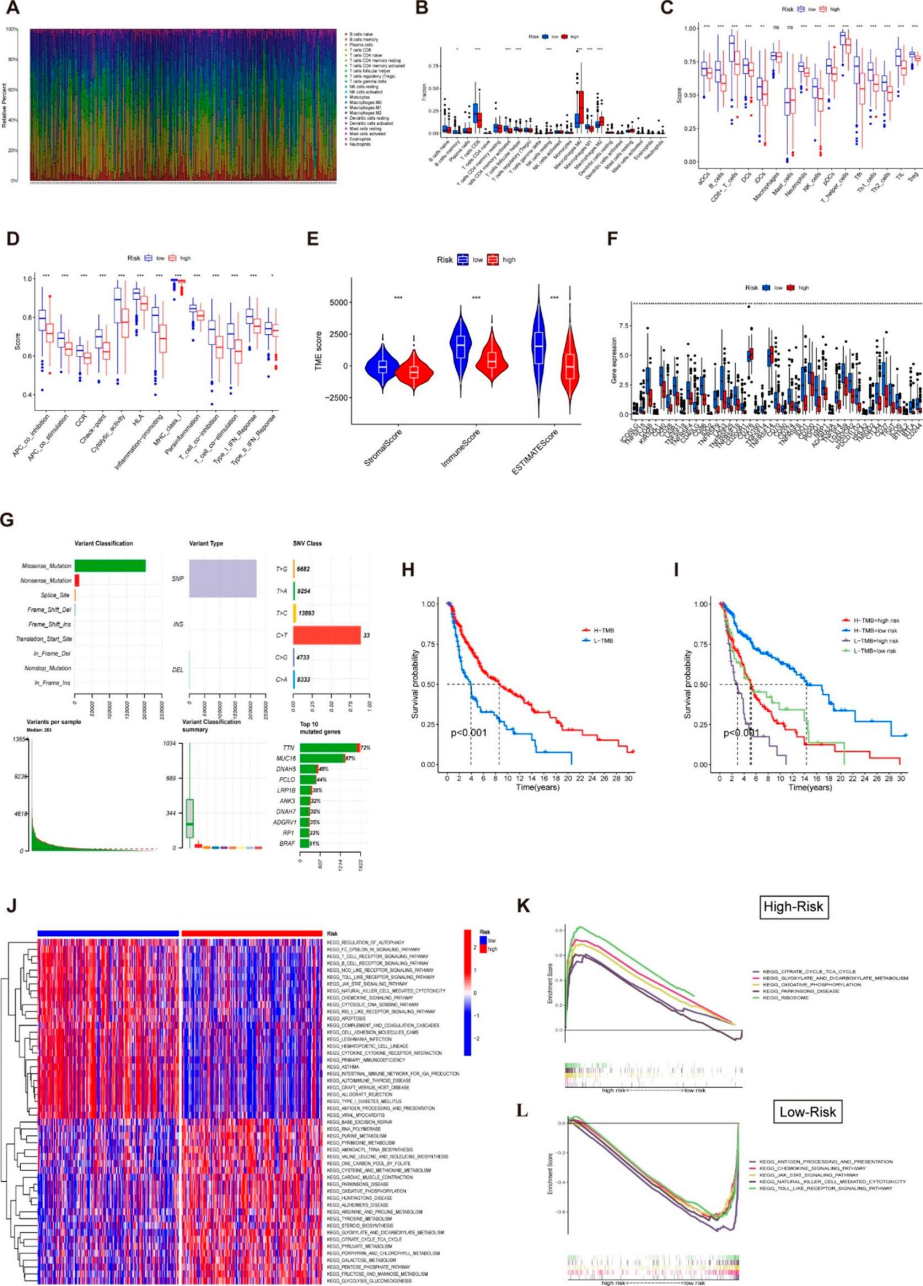
Correlation of necroptosis-related with the immune microenvironment. (**A**) The overview of 22 infiltrating immune cells in melanoma patients. (**B**) Boxplot showed the ratio difference of 22 types of immune cells in melanoma patients with high- and low-risk scores. The association between risk score and 16 types of immune cells (**C**) and 13 immune-related functions (**D**) in the low (blue box) and high-risk (red box) groups. We could see that immunological functions associated with necroptosis are more active in the low-risk group. (**E**) The comparison of immune-related scores between- high and low-risk groups. (**F**) The difference of 47 immune checkpoints genes expression in risk groups. (**G**) The overview of mutations in melanoma patients. (**H**) Kaplan-Meier survival curves for melanoma patients stratified by TMB. (**I**) Kaplan-Meier curves for melanoma patients stratified by both risk scores and TMB. (**J**) The results of GSVA analysis show the top 50 functional pathways with differences in high- and low-risk groups. (**K**) Representative enrichment plots generated by GSEA reveal that the high-risk was significantly associated with metabolism-related pathways, including the ribosome and the oxidative-phosphorylation signaling pathway. (**L**) Tumor-associated and immune response pathways were mainly enriched in the low-risk group, including the JAK-STAT signaling pathway and the Toll-like receptor signaling pathway. Note: *** P ≤ 0.001. ** P ≤ 0.01. * P ≤ 0.05

Figure 7 A demonstrates the overall profile of immune cells infiltration in melanoma samples. We found that memory B cells, CD8 T cells, activated memory CD4 cells, and M1 macrophages were more abundant in the low-risk group, whereas M0 and M2 macrophages were predominantly infiltrated in the high-risk groups (Fig. 7B). We then applied ssGSEA to examine the correlation between risk scores and immune cells and functions. Figure 7 C showed that the level of immune cell infiltration in the low-risk subgroups was generally higher than in the high-risk ones, except for mast cells and macrophages. Meanwhile, the high-risk subgroup also scored lower than the low-risk group on 13 types of immune-related functions (Fig. 7D). These findings suggest that immunological involvements associated with necroptosis are more active in the low-risk group. According to the ESTIMATE methodology, the high-risk populations had a considerably lower stromal score, immune score, and ESTIMATE score compared to the low-risk populations (Fig. 7E). Similarly, there was a discrepancy in the expression of genes involved in immune checkpoint-related genes between the two groups; with the low-risk group showing a trend toward elevated gene expression (Fig. 7F). Taken together, there were notable differences in immunological status between risk populations, which could shed light on the development of tumor immunotherapy for melanoma. Additionally, when it comes to predicting the efficacy of immunotherapy and chemotherapy, TMB is a crucial factor. More neo-antigens, which might be potential targets for immunotherapy and chemotherapy, are generated by patients with higher TMB. Figure 7G depicts the mutation profiles of each melanoma patient. A missense mutation was the primary single nucleotide mutation type and the top 5 most drastically mutated genes were TTN, MUC16, BRAF, DNAH5 and PCLO. Subsequently, we determined the TMB of each sample and found a correlation between TMB and OS; when comparing patients with different levels of TMB, those with higher levels had a longer overall survival rate (Fig. 7H). Notably, survival rates were better for patients with low-risk high-TMB than those with low-TMB high-risk (Fig. 7I). However, there was no discernible link between TMB and the risk score (Fig. S5).

### Functional signaling exploration of the model

GSVA and GSEA were utilized to explore differences in signaling pathways between the high- and low-risk populations, and representative pathways were selected for demonstration (Fig. 7J). The results of GSEA for high- and low-risk groups were available in Table S1 and S2. KEGG pathway analysis revealed that the lncRNAs of the high-risk group were primarily concentrated in the metabolic pathway, including the ribosome, oxidative phosphorylation, and glyoxylate and dicarboxylate metabolism pathways (Fig. 7K). Moreover, the JAK-STAT signaling pathway, the Toll-like receptor signaling pathway, and the antigen processing and presentation pathway were enriched in the low-risk group, indicating that low-risk patients are inextricably linked to the tumor-associated and immune response pathways (Fig. 7L). These findings suggest that the NRLs and signaling pathways differ in diverse risk populations, which may partially account for the significant prognostic differences between the two groups.

### Immunotherapy and drug sensitivity analysis

**Fig. 8:**
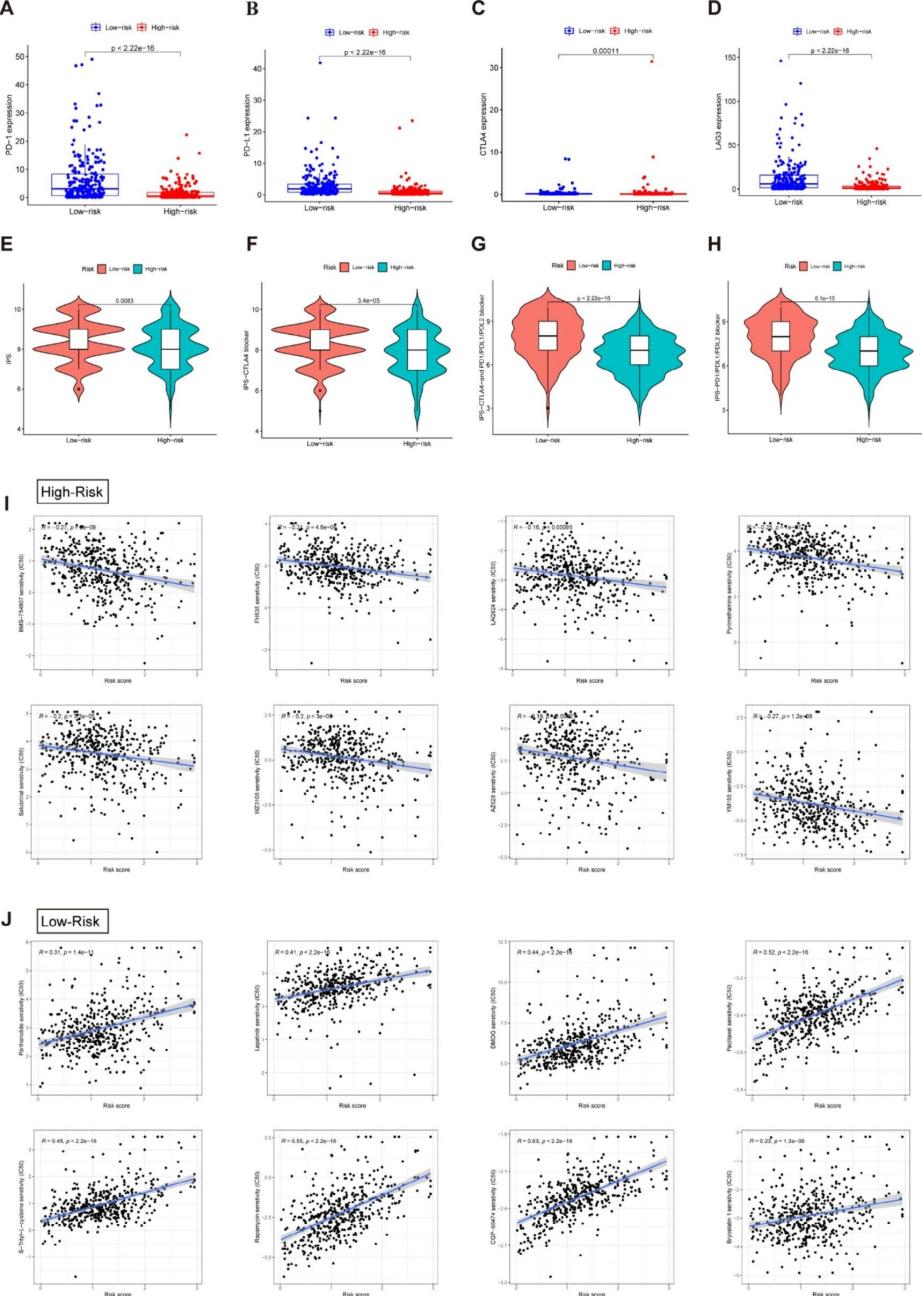
Prediction of immunotherapy response and chemotherapy drug sensitivity in melanoma patients. Comparison of expression levels of critical genes such as PD-1 (**A**), PD-L1 (**B**), CTLA4 (**C**) and LAG3 (**D**) for immune checkpoint inhibitors among distinct risk groups. (**E**-**H**) The estimation of immunotherapy response between high- and low-risk score groups. Orange represents the low-score group, and Cyan represents the high-score group. The thick line within the violin plot represents the median value. The inner box between the top and bottom represents the interquartile range. (**I**) Comparisons of IC_50_ for chemotherapeutics between two subgroups revealed that the high-risk group was more likely to benefit from BMS-754,807, FH535, LAQ824, etc. (**J**). The low-risk group was more sensitive to Parthenolide, Lapatinib, DMOG, etc.

We explored whether the risk model correlated with ICIs biomarkers and the corresponding IRs (PD-1, PD-L1, CTLA4 and LAG3) expressed more activity in the low-risk subgroup than in the high-risk group (Fig. 8A-D). Subsequently, we attempted to estimate how sensitive patients with varying risk scores are to ICIs. As illustrated in Fig. 8E-H, the patients in the low-risk subgroup had higher IPS, indicating that those patients may benefit from immunotherapy. Ultimately, we compared the efficacy of different chemotherapeutic drugs in the two risk groups. As observed in this study, patients with greater risk scores showed a lower IC_50_ for the following chemotherapeutic medications: BMS-754,807, FH535, LAQ824, Pyrimethamine, Salubrina, WZ3105, AZ628 and YM155 (Fig. 8I). Those in the low-risk group benefited from Parthenolide, Lapatinib, DMOG, S-Trityl-L-cysteine, Rapamycin, CGP-60,474 and Bryostatin 1 among others (Fig. 8J). Our findings support the application of NRLs to predict the immunotherapy response and chemotherapy drug sensitivity in melanoma patients, which facilitates the development of an individualized treatment strategy.

### Identifying AL162457.2 as a prognostic biomarker for melanoma

**Fig. 9:**
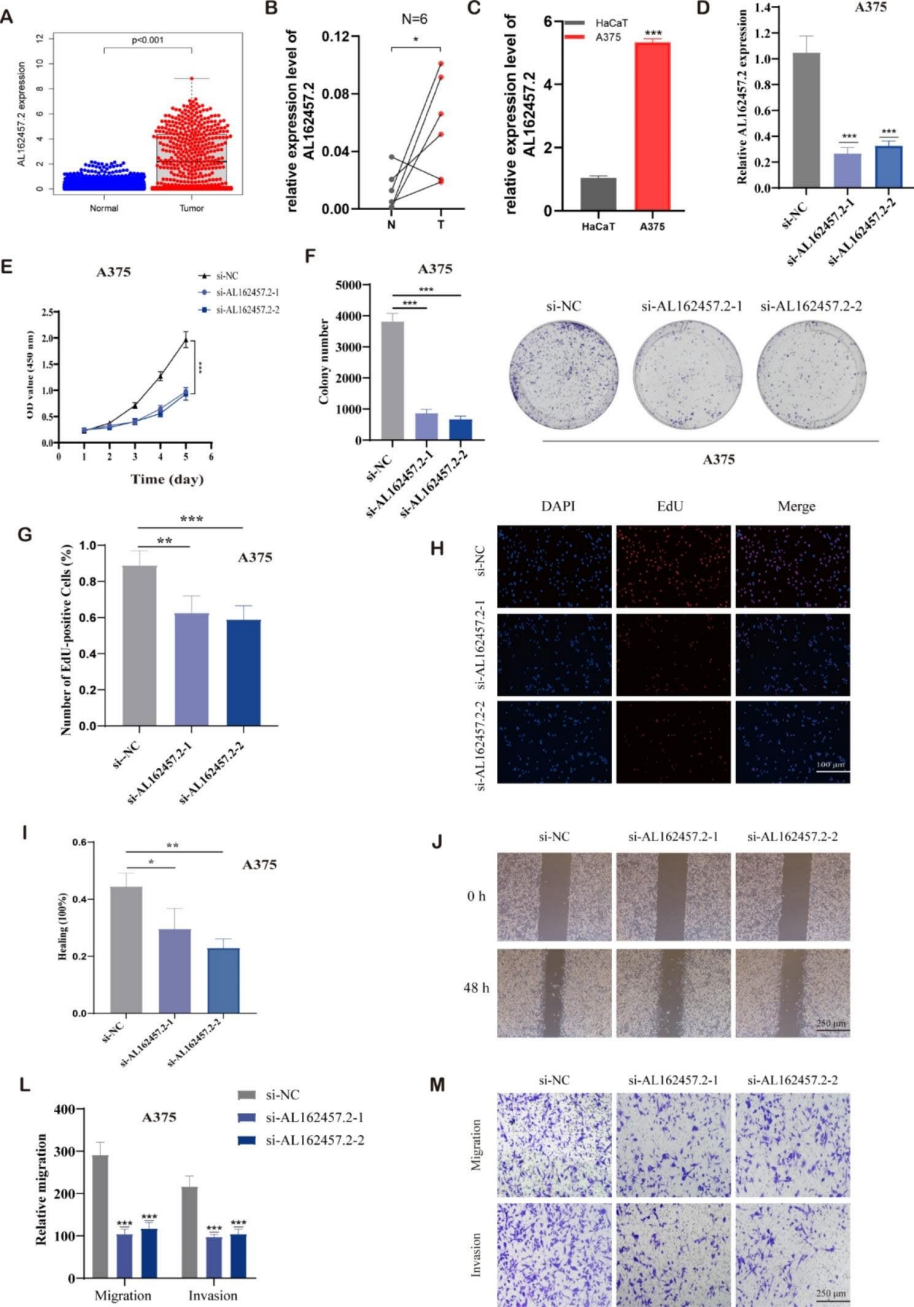
AL162457.2 inhibited the proliferation and migration of melanoma cells. (**A**) AL162457.2 was highly expressed in melanoma samples compared to the corresponding normal tissues of the GTEx-skin dataset as a control. (**B**) The relative expression of AL162457.2 in six pairs of melanoma and normal skin tissue samples. (**C**) The relative expression of AL162457.2 in melanoma cell lines (A375) with human keratinocyte cells (HaCaT). (**D**) The expression of AL162457.2 in HaCaT and A375 cells after transferring with si- AL162457.2 was determined by qRT-PCR. (**E**, **F**) The proliferation of A375 cells transfected with siRNA against AL162457.2 were measured using CCK-8 and colony formation assays. (**G**, **H**) EdU assay was utilized to detect the proliferative capacity of A375 cells after AL162457.2 downregulation. (**I**, **J**) Effects of AL162457.2 downregulation on melanoma cell migration, as evaluated by wound healing assay. (**L**, **M**) Transwell assays were applied to determine the migration and invasion capacity of A375 cells after AL162457.2 downregulation. Note: *** P ≤ 0.001. ** P ≤ 0.01. * P ≤ 0.05

As a further step toward identifying potential therapeutic hub lncRNAs in the NRLs signature, we examined the expression level and prognostic prediction value of single lncRNAs for melanoma using the TCGA dataset. We found that only AL162457.2 was significantly highly expressed in melanoma specimens (Fig. 9A), high-risk groups, and the C2 subtype. Further survival analysis demonstrated that patients with high AL162457.2 expression had remarkably shorter OS, suggesting that AL162457.2 was highly predictive of the prognosis of melanoma patients. Besides, AL162457.2 has the highest regression coefficient value in our prognostic model, indicating that it contributes the most to the model. So far, no relevant studies have been conducted on AL162457.2. Based on the above findings, we chose it to explore the potential expression mechanism and function in melanoma.

### Preliminary functional verification of AL162457.2

The expression of AL162457.2 in cancerous and adjacent tissues of six melanoma patients was examined by qRT-PCR, which confirmed that AL162457.2 was highly expressed in melanoma tissues. Similarly, AL162457.2 was significantly upregulated in A375 cell lines in comparison to HacaT as determined by PCR assay (Fig. 9B, C). The current work examined the efficacy of cell transfection by performing qRT-PCR and observed that AL162457.2 expression levels were drastically decreased after siRNA transfection (Fig. 9D). As expected, the CCK-8 assay and EdU assay confirmed that silencing AL162457.2 dramatically suppressed the proliferation ability of A375 cells (Fig. 9E, G-H). Also, interference with AL162457.2 expression inhibited colony formation (Fig. 9F). Besides, A375 cell migration, as well as invasiveness, also showed a decreased tendency after knockdown of AL162457.2 in comparison to those transfected with si-NC, measured by transwell assay and wound healing (Fig. 9I-M).

We further evaluated the molecular mechanisms involved in the AL162457.2-induced oncogenic phenotypic process. Gene ontology (GO) analysis showed that the differences between the high and low AL162457.2 expression groups were mainly focused on oxidative phosphorylation, respiratory electron transport chain, inner mitochondrial membrane protein complex and intermediate filament, suggesting the expression of AL162457.2 was related to mitochondrial metabolism (Figure S6). The GSEA indicated the high AL162457.2 group was mainly involved in oxidative phosphorylation (OXPHOS) and the lysosome signaling pathway. Accordingly, these findings suggest that these signaling pathways may be the underlying mechanisms that contribute to the adverse prognosis of melanoma. To further explore the target genes of AL162457.2, we employed co-expression analysis to calculate the correlation coefficients between lncRNAs and corresponding mRNAs, and screened 624 mRNAs with a significant positive correlation with AL162457.2. Then, we took intersections with NRGs and determined TIMM50 as the target gene of AL162457.2 (Table S3).

## Discussion

With the increased understanding of cell death mechanisms, non-apoptotic cell death is gradually becoming more apparent. Current research into non-apoptotic cell death focuses strongly on necroptosis, pyroptosis, and ferroptosis; each has distinct molecular characteristics [4]. Necroptosis is a significant cell death pattern that is triggered when cell apoptosis is blocked and is characterized by the activation of the caspase-independent signaling pathway; and is mainly mediated by the RIPK1/RIPK3/MLKL complex [4]. Necroptosis displays similar morphological characteristics to necrosis—for instance, plasma membrane disintegration, organelle swelling, cellular contents overflow, and loss of cell integrity [3]. In contrast to the absence of extravasation of cellular contents in apoptosis, necroptosis is a type of cytolytic death that regulates the occurrence and development of disease [3]. However, necroptosis may play two contrasting roles in cancer, inducing abnormal cell death and promoting cancer metastasis [3–5]. The downregulation of RIPK3 and MLKL may contribute to a poor prognosis in multiple cancers (such as breast cancer, melanoma, colorectal cancer and acute myeloid leukaemia) [3]. At the same time, the upregulation of RIPK3 or RIPK1 is involved in the occurrence and progression of glioma, pulmonary carcinoma, and pancreatic cancer [3]. Necroptosis can also accelerate metastasis via its participation in the process of inflammation or tumor cell-induced necrosis [22]. Therefore, there is a growing eagerness to further investigate the complex correlation between necroptosis and tumor generation and development.

LncRNAs are considered to be RNA transcripts longer than 200 nucleotides in length rather than encoding peptides [23]. They can be distinguished from small non-coding RNAs based on their length. LncRNAs are implicated in numerous processes, including mammalian development, inflammatory diseases, and cancers, and can serve as a biomarker of diseases [24]. Abnormal expression of lncRNAs may be relevant to tumorigenesis and prognosis [25]. There is increasing evidence that NRLs are connected to the initiation and progression of malignancies. In the case of melanoma, however, there is still insufficient evidence in this area.

We first identified NRLs by performing univariate and multifactorial Cox regression analysis. The following seven NRLs were pinpointed as having a vital prognostic influence on melanoma patients through LASSO regression: EBLN3P, AC093010.2, LINC01871, IRF2-DT, AL162457.2, AC242842.1, and HLA-DQB1-AS1. We categorized patients into two distinct risk groups according to the median risk value calculated by the risk formula. The goal of this study is to identify potential characteristics and optimal treatment options for different risk groups through the constructed lncRNA model. We carried out a GSEA so that a deeper comprehension of the potential functional enrichment pathway of the model could be identified. The GSEA results revealed that the JAK-STAT signaling pathway, the Toll-like receptor signaling pathway and the natural killer cell-mediated cytotoxicity were enriched in the low-risk population. The JAK-STAT pathway plays a crucial role in cell division and differentiation, organ growth and development, and immunological homeostasis; additionally, it induces the expression of multifarious pivotal mediators of cancer and inflammation [26]. The Toll-like receptor (TLR) pathway is instrumental in controlling inflammasome activity, innate immunity and tumor progression [27]. Furthermore, the TLR pathway is associated with necroptosis. TRIF(Toll/IL-1R domain-containing adapter-inducing interferon-β) is involved in the RIPK1/RIPK3 complex and activates the MLKL- associated necroptotic death pathway [27]. Combining immunotherapy with the above pathways is a practical approach for observing the desirable effects in low-risk populations. Meanwhile, ribosome, citrate cycle (TCA cycle), oxidative phosphorylation, and glyoxylate-dicarboxylate metabolism have significant associations with high-risk populations. It can be speculated that metabolism-related treatments are beneficial for high-risk groups.

To investigate the immunomodulatory mechanisms in this prognostic model, immunity-related algorithms were conducted. Following our findings, the low-risk subgroup exhibited more significant infiltration of immune cells, immune-linked pathways, immune checkpoint expression and tumor microenvironment components, which may provide an explanation for better prognosis of the low-risk populations. Simultaneously, we predicted the immunotherapy responsiveness of distinct risk subgroups according to the TMB and IPS. TMB, a measure of the number of cancer mutations, was initially identified as a potential biomarker for ICIs in melanoma[28]. Clinically, higher TMB corresponds with objective responses to PD-1/PD-L1 inhibitors as it generates more neo-antigens, which increases the likelihood of T-cell recognition [29, 30]. Considering these results, low-risk patients with high TMB exhibit better clinical outcomes than those with low TMB. ICIs have revolutionized the treatment of patients with advanced-stage melanoma[31]. The low-risk group would respond better to immunotherapy, indicating that our signature could serve as a potential index for determining the effectiveness of immunotherapy in melanoma patients. The issues of selecting the optimal combination of ICIs, predicting and managing the toxic effects of different combinations, screening for sensitive and specific tumor markers, and improving the 5-year survival rate of patients remain major challenges in melanoma treatment. By comparing their susceptibility to a range of chemotherapy drugs, we concluded that the different risk groups have varying sensitivity to these drugs. We predicted the sensitivity of some compounds in the melanoma population, which help to guide the clinical treatment of melanoma. Moreover, future research could involve further subdivisions of the population (according to various characteristics) and selecting suitable drugs for these subgroups.

Among these seven candidate lncRNAs constituting the prognostic model, we observed that AL162457.2 was significantly highly expressed in melanoma high-risk population and C2 subtype. However, no studies have been reported on the biological functions associated with AL162457.2. We finally explored the functional phenotype of AL162457.2 using preliminary experiments. High expression of AL162457.2 predicted decreased OS in melanoma. We verified the high relative expression of AL162457.2 in melanoma tissues or cell lines, and the results were consistent with the data in the public databases. Of note, suppressing AL162457.2 inhibited melanoma cell proliferation and migration in vitro. Based on the studies, AL162457.2 could serve as an underlying therapeutic target of melanoma patients. The AL162457.2 high and low expression groups were compared using GSEA to determine which biochemical pathways were significantly enriched in either group. The results of GSEA identified 10 AL162457.2-associated significantly enriched pathways and the oxidative phosphorylation (OXPHOS) was part of the activated signaling pathway. To our knowledge, the OXPHOS metabolic pathway is responsible for ATP production by ferrying electrons to a chain of transmembrane protein complexes in the inner mitochondrial membrane [32]. The upregulation of OXPHOS in certain types of cancer may make them vulnerable to OXPHOS inhibition. Consequently, we speculated that AL162457.2 is involved in the oxidative phosphorylation pathway to drive melanoma cells growth, invasion and migration. However, the crosstalk and mechanism of the above bioinformatics prediction need verification with well-designed experiments.

Increasing evidence has shown that lncRNAs can directly bind to mRNA to affect mRNA translation and act as decoys for miRNAs and proteins [21]. According to bioinformatics analysis, we found that the expression of AL162457.2 was most positively and significantly related to the mRNA expression of TIMM50 (Pearson correlation coefficient: 0.467). TIMM50 (Translocase of the inner mitochondrial membrane 50), also called TIM50, is the receptor subunit that directs pre-protein transportation from the outer mitochondrial membrane (TOM complex) to the inner mitochondrial membrane (TIM23 complex) [33, 34]. According to previous literature, TIMM50 may function as an oncogenetic protein in breast cancer. Sankala et al. revealed that the expression of TIMM50 might be upregulated by overexpressing a mutant of P53, thus causing breast cancer cell growth and chemoresistance [33, 35]. Zhang et al. demonstrated that TIMM50 facilitated tumor proliferation and invasion of non-small cell lung cancer (NSCLC) through enhancing phosphorylation of its downstream ERK/P90RSK signaling pathway, and speculated that TIMM50 might be a useful prognosis marker for NSCLC patients [36]. In the future, we will investigate the mechanism of AL162457.2’s targeted regulation of TIMM50 and contribute to revealing the lncRNA-mRNA network relationship of AL162457.2 in melanoma.

Although we have constructed a predictive model based on seven necroptosis-associated lncRNAs in melanoma patients, there are still some limitations to consider. Firstly, only the TCGA database was provided as the basis for the original dataset, for setting up the lncRNA-related model, given the inherent limitation of insufficient samples including lncRNA expression profiles in clinical databases. We need to verify our risk model on external datasets and melanoma samples to ensure its accuracy and consistency. Additionally, our investigations of AL162457.2’s functional phenotype have been rudimentary and the mechanism leading to AL162457.2 upregulation in melanoma remains to be fully illustrated. Therefore, further experiments are needed to identify AL162457.2 specific roles and mechanisms in melanoma.

## Conclusions

Overall, we conducted comprehensive bioinformatics analysis and identified a predictive model for melanoma prognosis based on seven NRLs. As we learned, this is the first predictive model for melanoma to target NRLs. Our signature will contribute to greater comprehension of the specific effects of NRLs in melanoma. We hope to provide creative insights for melanoma treatment strategies.

### Electronic supplementary material

Below is the link to the electronic supplementary material.

Supplementary Material 1

Supplementary Material 2

Supplementary Material 3

Supplementary Material 4

## Data Availability

The datasets used and/or analysed during the current study are available from the corresponding author on reasonable request.

## Abbreviations

NRLs: Necroptosis-Related LncRNAs
TCGA: The Cancer Genome Atlas
LASSO: Least absolute shrinkage and selection operator
LncRNA: Long non-coding RNA
OS: Overall survival
RIPK1/3: Receptor-interacting protein kinase1/3
MLKL: Mixed Lineage Kinase domain-Like protein
TNFα: Tumor Necrosis Factorα
TNFR: Tumor Necrosis Factor Receptor
IAP: Inhibitor of Apoptosis Proteins
TMB: Tumor mutational burden
NRGs: Necroptosis-Related genes
K-M: Kaplan–Meier
t-SNE: t-distributed stochastic neighbor embedding
PCA: Principal component analysis
ROC: Receiver operating characteristic
MAF: Mutation Annotation Format
GSVA: Gene Set Variation Analysis
ssGSEA: Single-sample gene set enrichment analysis
SKCM: Skin Cutaneous Melanoma
GSEA: Gene set enrichment analysis
KEGG: Kyoto Encyclopedia of Genes and Genomes
IRs: Inhibitory receptors
PD1: Programmed cell death protein 1
PDL1: Programmed cell death-Ligand 1
CTLA4: Cytotoxic T lymphocyte-associated protein 4
LAG3: Lymphocyte-activation-gene-3
IPS: Immunophenoscore
ICIs: Immune checkpoint inhibitors
TCIA: The Cancer Immunome Atlas
MHC: Major histocompatibility complexes
IC_50_: Half maximal inhibitory concentration
HaCaT: Human keratinocyte cells
FBS: Fetal bovine serum
siRNA: Small interfering RNA
NC: Negative control
qRT-PCR: RNA Extraction and Quantitative Real-time PCR
CCK-8: Cell Counting Kit-8
AUC: Area under the curve
GO: Gene Ontology
TLR: Toll-like receptor
TRIF: Toll/IL-1R domain-containing adapter-inducing interferon-β
OXPHOS: Oxidative phosphorylation
