# Supplementary material for: Necroptosis-related LncRNAs in skin cutaneous melanoma: evaluating prognosis, predicting immunity, and guiding therapy
Source: BMC Cancer. 2023 Aug 14;23:752. doi: 10.1186/s12885-023-11246-x (PMC10424397; doi:10.1186/s12885-023-11246-x)
Supplement: Supplementary file 1 — Supplementary Material 1 [file 12885_2023_11246_MOESM1_ESM.docx]

**
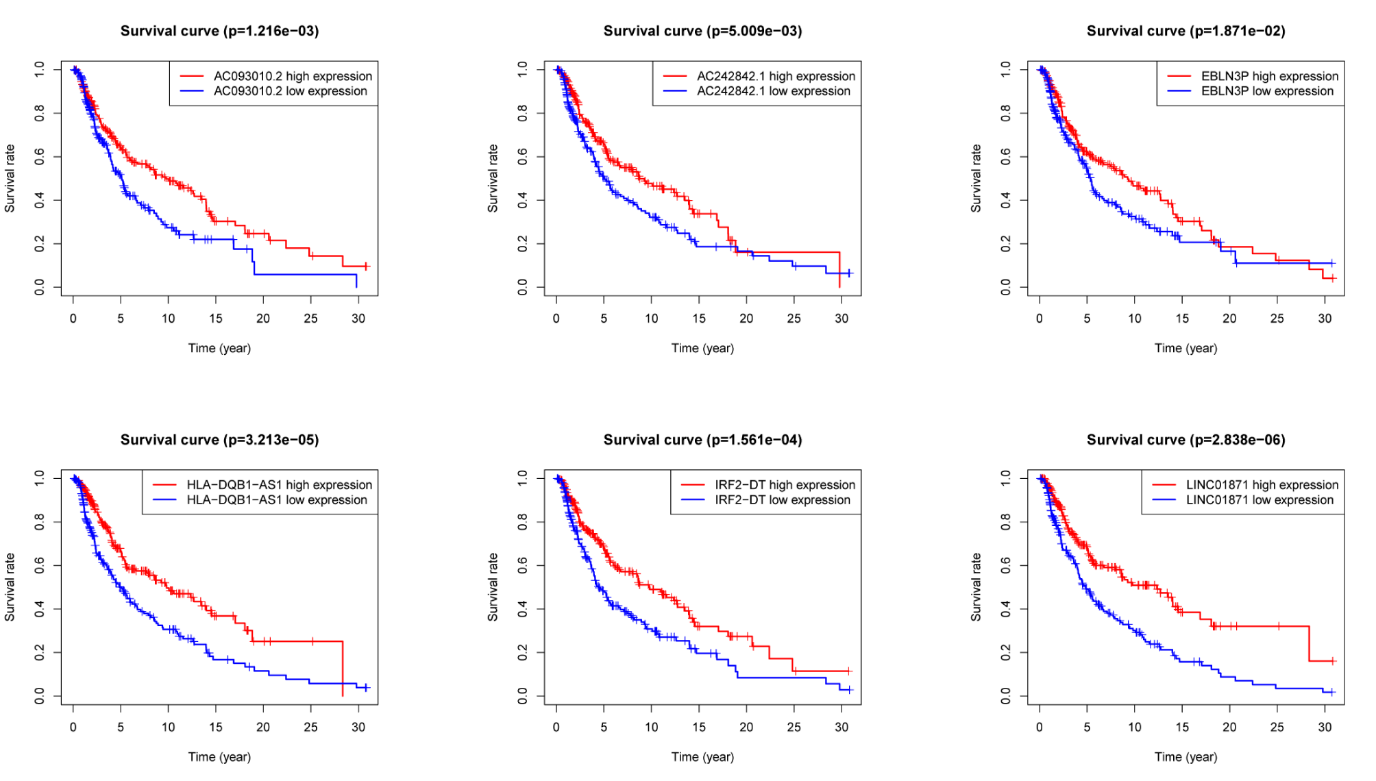
**

**Figure S1** Survival curve of melanoma patients with high and low model lncRNA expression (including EBLN3P, AC093010.2, LINC01871, IRF2-DT, AC242842.1, HLA-DQB1-AS1).


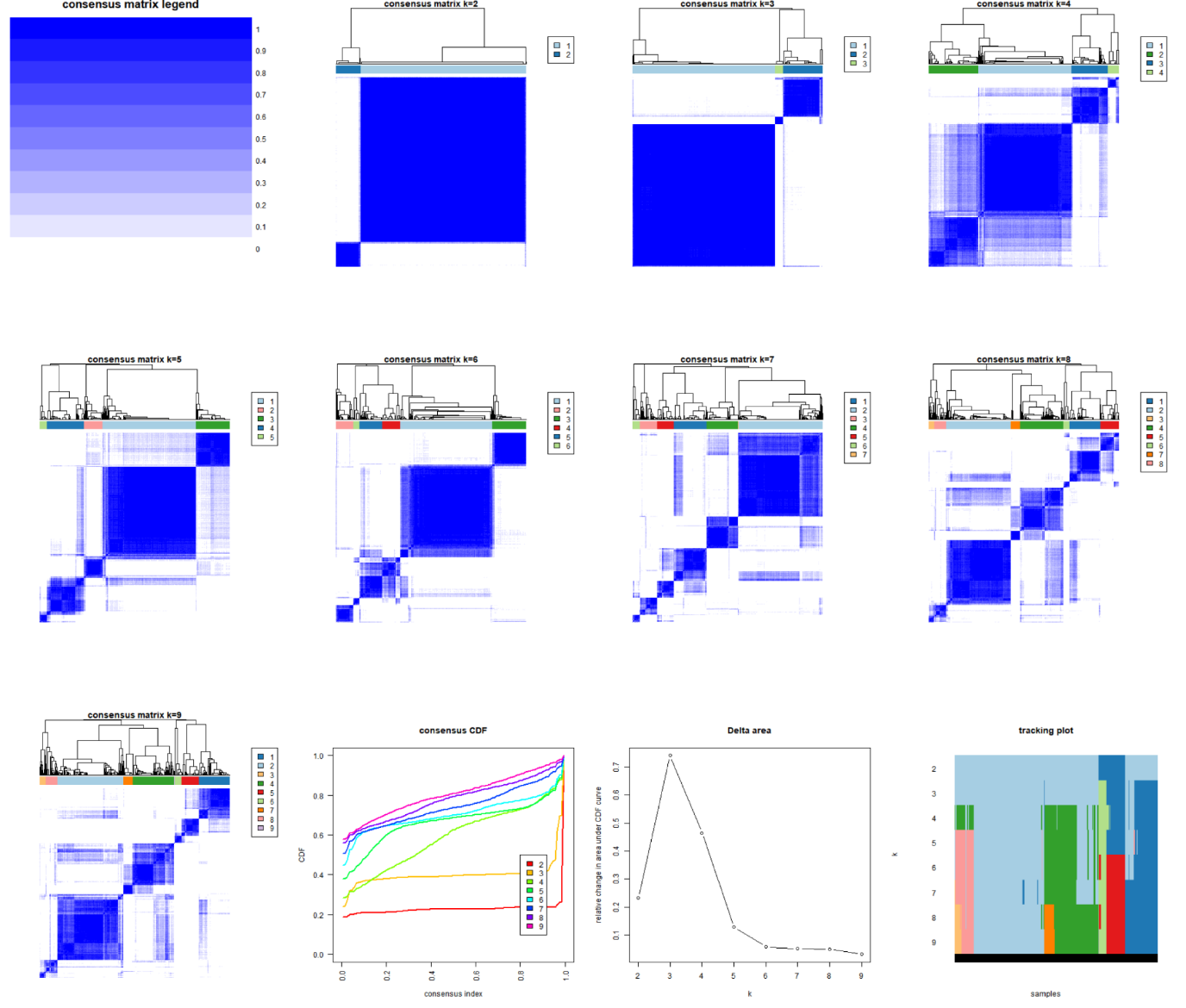


**Figure S2** Consensus clustering analysis process for risk lncRNAs.


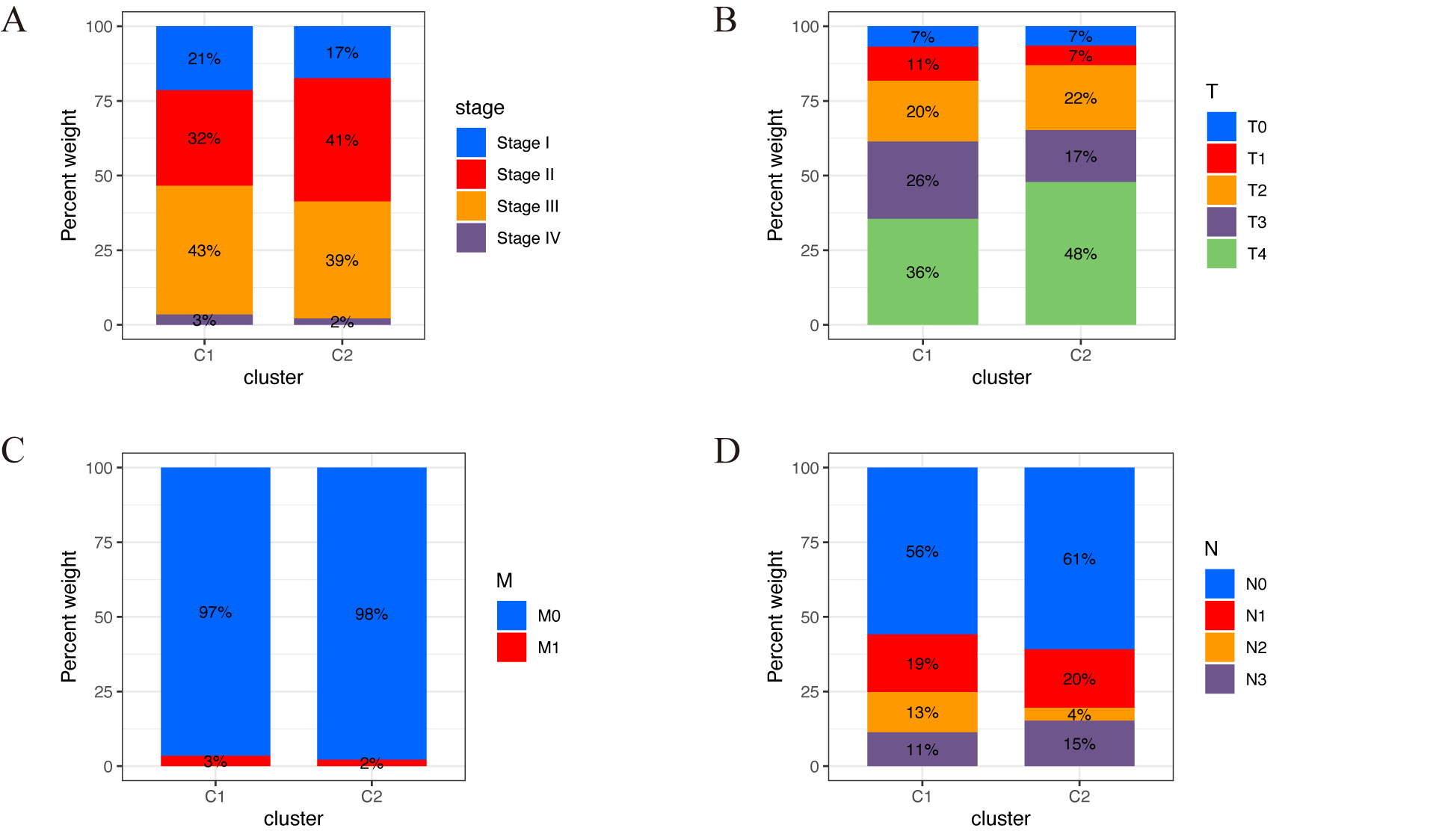


**Figure S3** Differences in clinical features for different melanoma clusters. The proportion of patients with different stages (A), T stage (B), M stage (C), N stage (D) in the two clusters.


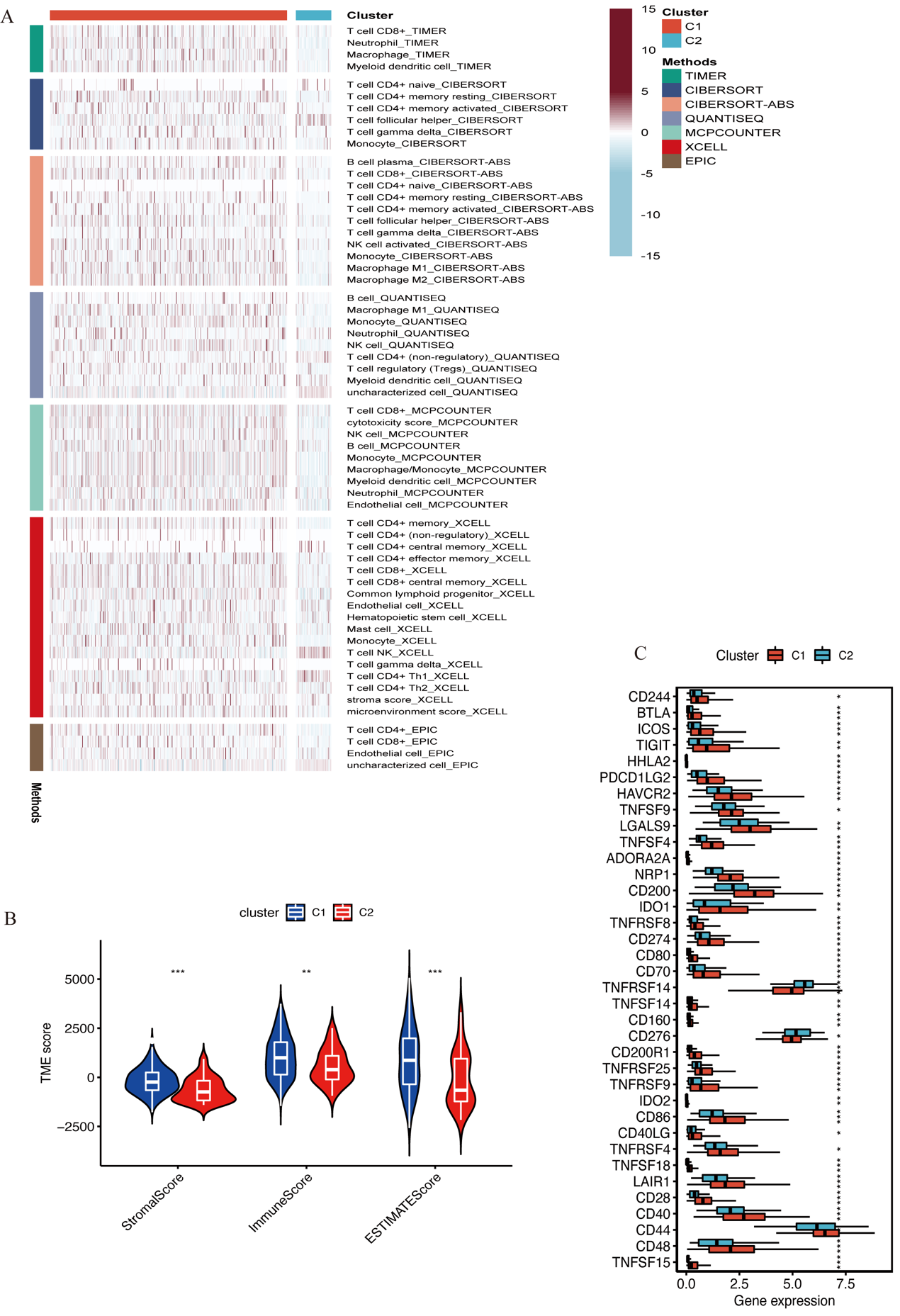


**Figure S4** Immune signatures of six necroptosis-related lncRNAs (NRLs) melanoma clusters. (A) The heatmap of immune cells in clusters based on different platforms. (B) The comparison of immune-related scores between C1 and C2. (C) Differential expression of immune checkpoint-related genes in different clusters.


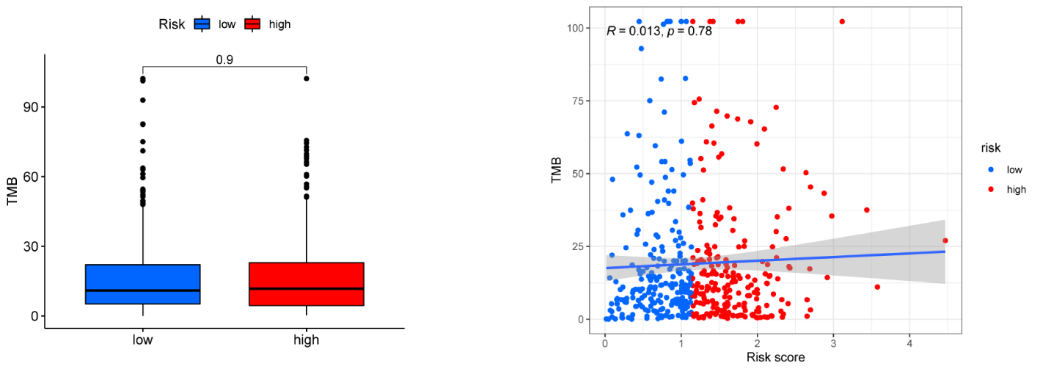


**Figure S5** No significant differences in TMB values were observed between high and low risk groups.


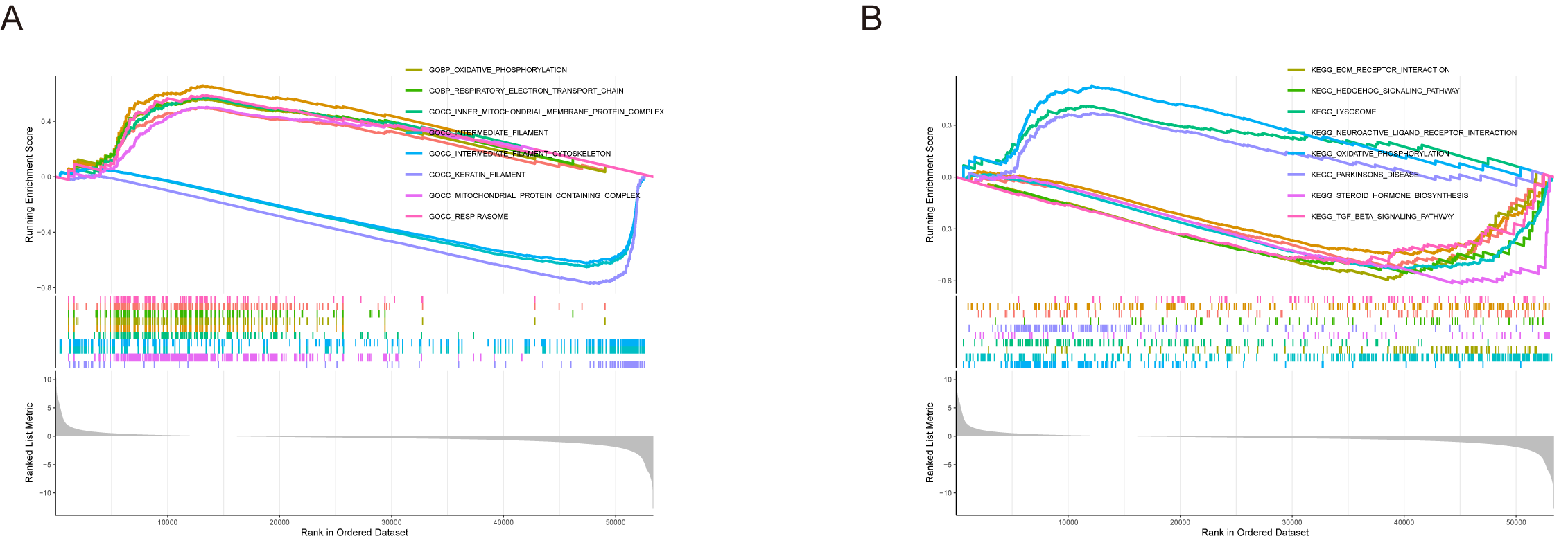


**Figure S6** (A) The results of GO analysis revealed significant functional differences between the high and low AL162457.2 expression groups. (B) Significantly enriched pathways in the high and low AL162457.2 expression groups. The part of the curve with the peak at the top indicates a positive correlation between expression levels and pathway activity, and vice versa.
