# Supplementary material for: Necroptosis-related LncRNAs in skin cutaneous melanoma: evaluating prognosis, predicting immunity, and guiding therapy
Source: BMC Cancer. 2023 Aug 14;23:752. doi: 10.1186/s12885-023-11246-x (PMC10424397; doi:10.1186/s12885-023-11246-x)
Supplement: Supplementary file 2 — Supplementary Material 2 [file 12885_2023_11246_MOESM2_ESM.docx]

**Table S1. GSEA of high risk.**

| **Name** | **ES** | **NES** | **NOM *p-val*** | **FDR *q-val*** |
| --- | --- | --- | --- | --- |
| KEGG_OXIDATIVE_PHOSPHORYLATION | 0.69 | 2.08 | 0.000 | 0.007 |
| KEGG_GLYOXYLATE_AND_DICARBOXYLATE_METABOLISM | 0.73 | 1.93 | 0.000 | 0.034 |
| KEGG_PARKINSONS_DISEASE | 0.61 | 1.91 | 0.006 | 0.029 |
| KEGG_RIBOSOME | 0.83 | 1.87 | 0.006 | 0.033 |
| KEGG_ALZHEIMERS_DISEASE | 0.47 | 1.75 | 0.016 | 0.094 |
| KEGG_HUNTINGTONS_DISEASE | 0.46 | 1.74 | 0.014 | 0.083 |
| KEGG_CITRATE_CYCLE_TCA_CYCLE | 0.61 | 1.69 | 0.036 | 0.116 |
| KEGG_RNA_POLYMERASE | 0.55 | 1.68 | 0.031 | 0.111 |
| KEGG_PYRIMIDINE_METABOLISM | 0.44 | 1.59 | 0.048 | 0.183 |
| KEGG_AMINOACYL_TRNA_BIOSYNTHESIS | 0.55 | 1.58 | 0.048 | 0.178 |
| KEGG_PENTOSE_PHOSPHATE_PATHWAY | 0.52 | 1.54 | 0.042 | 0.203 |
| KEGG_FRUCTOSE_AND_MANNOSE_METABOLISM | 0.48 | 1.54 | 0.046 | 0.195 |
| KEGG_PURINE_METABOLISM | 0.36 | 1.51 | 0.039 | 0.210 |
| KEGG_STEROID_BIOSYNTHESIS | 0.6 | 1.5 | 0.075 | 0.209 |
| KEGG_BASE_EXCISION_REPAIR | 0.5 | 1.48 | 0.096 | 0.224 |
| KEGG_BIOSYNTHESIS_OF_UNSATURATED_FATTY_ACIDS | 0.52 | 1.46 | 0.099 | 0.233 |
| KEGG_GLYCOLYSIS_GLUCONEOGENESIS | 0.43 | 1.43 | 0.087 | 0.252 |
| KEGG_VIBRIO_CHOLERAE_INFECTION | 0.4 | 1.42 | 0.069 | 0.244 |
| KEGG_AMINO_SUGAR_AND_NUCLEOTIDE_SUGAR_METABOLISM | 0.43 | 1.41 | 0.085 | 0.246 |
| KEGG_GALACTOSE_METABOLISM | 0.47 | 1.41 | 0.079 | 0.236 |
| KEGG_CARDIAC_MUSCLE_CONTRACTION | 0.39 | 1.37 | 0.098 | 0.275 |
| KEGG_DNA_REPLICATION | 0.53 | 1.34 | 0.220 | 0.292 |
| KEGG_ONE_CARBON_POOL_BY_FOLATE | 0.48 | 1.32 | 0.155 | 0.314 |
| KEGG_PYRUVATE_METABOLISM | 0.41 | 1.32 | 0.153 | 0.307 |
| KEGG_TERPENOID_BACKBONE_BIOSYNTHESIS | 0.52 | 1.26 | 0.242 | 0.373 |
| KEGG_PORPHYRIN_AND_CHLOROPHYLL_METABOLISM | 0.39 | 1.22 | 0.217 | 0.417 |
| KEGG_BLADDER_CANCER | 0.35 | 1.19 | 0.203 | 0.448 |
| KEGG_ARGININE_AND_PROLINE_METABOLISM | 0.33 | 1.16 | 0.249 | 0.487 |
| KEGG_GLYCEROPHOSPHOLIPID_METABOLISM | 0.27 | 1.11 | 0.272 | 0.554 |
| KEGG_GLUTATHIONE_METABOLISM | 0.33 | 1.11 | 0.314 | 0.548 |
| KEGG_CYSTEINE_AND_METHIONINE_METABOLISM | 0.33 | 1.09 | 0.340 | 0.566 |
| KEGG_TYROSINE_METABOLISM | 0.33 | 1.07 | 0.352 | 0.571 |
| KEGG_LYSINE_DEGRADATION | 0.32 | 1.07 | 0.378 | 0.564 |
| KEGG_GLYCEROLIPID_METABOLISM | 0.29 | 1.05 | 0.382 | 0.584 |
| KEGG_LYSOSOME | 0.31 | 1.04 | 0.401 | 0.577 |
| KEGG_GLYCOSYLPHOSPHATIDYLINOSITOL_GPI_ANCHOR_BIOSYNTHESIS | 0.37 | 1.04 | 0.375 | 0.570 |
| KEGG_PHENYLALANINE_METABOLISM | 0.36 | 1.01 | 0.450 | 0.610 |
| KEGG_SELENOAMINO_ACID_METABOLISM | 0.3 | 0.94 | 0.516 | 0.712 |
| KEGG_SPHINGOLIPID_METABOLISM | 0.29 | 0.93 | 0.560 | 0.723 |
| KEGG_NUCLEOTIDE_EXCISION_REPAIR | 0.3 | 0.92 | 0.520 | 0.725 |
| KEGG_MISMATCH_REPAIR | 0.35 | 0.89 | 0.565 | 0.755 |
| KEGG_GLYCOSPHINGOLIPID_BIOSYNTHESIS_LACTO_AND_NEOLACTO_SERIES | 0.28 | 0.89 | 0.623 | 0.748 |
| KEGG_THYROID_CANCER | 0.27 | 0.88 | 0.639 | 0.739 |
| KEGG_METABOLISM_OF_XENOBIOTICS_BY_CYTOCHROME_P450 | 0.31 | 0.87 | 0.632 | 0.736 |
| KEGG_ADHERENS_JUNCTION | 0.25 | 0.87 | 0.633 | 0.725 |
| KEGG_OTHER_GLYCAN_DEGRADATION | 0.33 | 0.86 | 0.624 | 0.729 |
| KEGG_HOMOLOGOUS_RECOMBINATION | 0.32 | 0.85 | 0.611 | 0.735 |
| KEGG_PROPANOATE_METABOLISM | 0.26 | 0.77 | 0.708 | 0.858 |
| KEGG_PENTOSE_AND_GLUCURONATE_INTERCONVERSIONS | 0.26 | 0.75 | 0.780 | 0.861 |
| KEGG_GLYCINE_SERINE_AND_THREONINE_METABOLISM | 0.24 | 0.75 | 0.830 | 0.845 |
| KEGG_BASAL_CELL_CARCINOMA | 0.22 | 0.75 | 0.813 | 0.836 |
| KEGG_RENIN_ANGIOTENSIN_SYSTEM | 0.25 | 0.68 | 0.909 | 0.920 |
| KEGG_DRUG_METABOLISM_CYTOCHROME_P450 | 0.21 | 0.57 | 0.983 | 0.991 |
| KEGG_RETINOL_METABOLISM | 0.19 | 0.49 | 0.996 | 1.000 |
| KEGG_STEROID_HORMONE_BIOSYNTHESIS | 0.15 | 0.43 | 1.000 | 0.995 |
