# Supplementary material for: Necroptosis-related LncRNAs in skin cutaneous melanoma: evaluating prognosis, predicting immunity, and guiding therapy
Source: BMC Cancer. 2023 Aug 14;23:752. doi: 10.1186/s12885-023-11246-x (PMC10424397; doi:10.1186/s12885-023-11246-x)
Supplement: Supplementary file 3 — Supplementary Material 3 [file 12885_2023_11246_MOESM3_ESM.docx]

**Table S2. GSEA of low risk.**

| **Name** | **ES** | **NES** | **NOM *p-val*** | **FDR *q-val*** |
| --- | --- | --- | --- | --- |
| KEGG_JAK_STAT_SIGNALING_PATHWAY | -0.65 | -2.31 | 0.000 | 0.000 |
| KEGG_TYPE_I_DIABETES_MELLITUS | -0.88 | -2.28 | 0.000 | 0.000 |
| KEGG_CHEMOKINE_SIGNALING_PATHWAY | -0.64 | -2.26 | 0.000 | 0.000 |
| KEGG_VIRAL_MYOCARDITIS | -0.7 | -2.24 | 0.000 | 0.000 |
| KEGG_LEISHMANIA_INFECTION | -0.78 | -2.23 | 0.000 | 0.000 |
| KEGG_NATURAL_KILLER_CELL_MEDIATED_CYTOTOXICITY | -0.66 | -2.23 | 0.000 | 0.000 |
| KEGG_ANTIGEN_PROCESSING_AND_PRESENTATION | -0.74 | -2.22 | 0.000 | 0.000 |
| KEGG_CYTOKINE_CYTOKINE_RECEPTOR_INTERACTION | -0.64 | -2.21 | 0.000 | 0.000 |
| KEGG_AUTOIMMUNE_THYROID_DISEASE | -0.81 | -2.2 | 0.000 | 0.000 |
| KEGG_TOLL_LIKE_RECEPTOR_SIGNALING_PATHWAY | -0.64 | -2.18 | 0.000 | 0.000 |
| KEGG_HEMATOPOIETIC_CELL_LINEAGE | -0.72 | -2.12 | 0.002 | 0.001 |
| KEGG_CELL_ADHESION_MOLECULES_CAMS | -0.63 | -2.08 | 0.002 | 0.002 |
| KEGG_NOD_LIKE_RECEPTOR_SIGNALING_PATHWAY | -0.67 | -2.07 | 0.000 | 0.002 |
| KEGG_INTESTINAL_IMMUNE_NETWORK_FOR_IGA_PRODUCTION | -0.82 | -2.07 | 0.004 | 0.002 |
| KEGG_ASTHMA | -0.83 | -2.07 | 0.002 | 0.002 |
| KEGG_GRAFT_VERSUS_HOST_DISEASE | -0.87 | -2.04 | 0.002 | 0.003 |
| KEGG_SYSTEMIC_LUPUS_ERYTHEMATOSUS | -0.59 | -2.03 | 0.002 | 0.003 |
| KEGG_T_CELL_RECEPTOR_SIGNALING_PATHWAY | -0.65 | -2.03 | 0.002 | 0.003 |
| KEGG_RIG_I_LIKE_RECEPTOR_SIGNALING_PATHWAY | -0.61 | -2.03 | 0.000 | 0.003 |
| KEGG_FC_GAMMA_R_MEDIATED_PHAGOCYTOSIS | -0.55 | -2.02 | 0.002 | 0.003 |
| KEGG_ALLOGRAFT_REJECTION | -0.89 | -2.01 | 0.002 | 0.003 |
| KEGG_APOPTOSIS | -0.57 | -1.94 | 0.002 | 0.008 |
| KEGG_B_CELL_RECEPTOR_SIGNALING_PATHWAY | -0.62 | -1.93 | 0.006 | 0.008 |
| KEGG_PRIMARY_IMMUNODEFICIENCY | -0.81 | -1.9 | 0.004 | 0.011 |
| KEGG_FC_EPSILON_RI_SIGNALING_PATHWAY | -0.52 | -1.89 | 0.000 | 0.012 |
| KEGG_MAPK_SIGNALING_PATHWAY | -0.45 | -1.85 | 0.000 | 0.017 |
| KEGG_LEUKOCYTE_TRANSENDOTHELIAL_MIGRATION | -0.52 | -1.85 | 0.002 | 0.017 |
| KEGG_VASCULAR_SMOOTH_MUSCLE_CONTRACTION | -0.48 | -1.85 | 0.000 | 0.017 |
| KEGG_SNARE_INTERACTIONS_IN_VESICULAR_TRANSPORT | -0.54 | -1.78 | 0.002 | 0.031 |
| KEGG_COMPLEMENT_AND_COAGULATION_CASCADES | -0.61 | -1.78 | 0.002 | 0.031 |
| KEGG_CYTOSOLIC_DNA_SENSING_PATHWAY | -0.56 | -1.77 | 0.008 | 0.031 |
| KEGG_ENDOCYTOSIS | -0.42 | -1.71 | 0.004 | 0.050 |
| KEGG_ADIPOCYTOKINE_SIGNALING_PATHWAY | -0.47 | -1.71 | 0.002 | 0.048 |
| KEGG_LONG_TERM_POTENTIATION | -0.47 | -1.7 | 0.008 | 0.051 |
| KEGG_P53_SIGNALING_PATHWAY | -0.47 | -1.67 | 0.028 | 0.065 |
| KEGG_GNRH_SIGNALING_PATHWAY | -0.41 | -1.66 | 0.004 | 0.066 |
| KEGG_LONG_TERM_DEPRESSION | -0.43 | -1.64 | 0.020 | 0.072 |
| KEGG_VEGF_SIGNALING_PATHWAY | -0.41 | -1.64 | 0.008 | 0.071 |
| KEGG_PHOSPHATIDYLINOSITOL_SIGNALING_SYSTEM | -0.43 | -1.61 | 0.030 | 0.084 |
| KEGG_NON_SMALL_CELL_LUNG_CANCER | -0.46 | -1.6 | 0.025 | 0.092 |
| KEGG_TYPE_II_DIABETES_MELLITUS | -0.45 | -1.59 | 0.017 | 0.094 |
| KEGG_REGULATION_OF_AUTOPHAGY | -0.51 | -1.56 | 0.026 | 0.108 |
| KEGG_REGULATION_OF_ACTIN_CYTOSKELETON | -0.4 | -1.56 | 0.024 | 0.107 |
| KEGG_UBIQUITIN_MEDIATED_PROTEOLYSIS | -0.43 | -1.56 | 0.046 | 0.105 |
| KEGG_ACUTE_MYELOID_LEUKEMIA | -0.46 | -1.55 | 0.047 | 0.108 |
| KEGG_PATHWAYS_IN_CANCER | -0.37 | -1.53 | 0.029 | 0.122 |
| KEGG_PANCREATIC_CANCER | -0.45 | -1.53 | 0.060 | 0.121 |
| KEGG_NEUROTROPHIN_SIGNALING_PATHWAY | -0.4 | -1.52 | 0.042 | 0.120 |
| KEGG_PANTOTHENATE_AND_COA_BIOSYNTHESIS | -0.54 | -1.52 | 0.034 | 0.118 |
| KEGG_INSULIN_SIGNALING_PATHWAY | -0.37 | -1.5 | 0.028 | 0.129 |
| KEGG_NEUROACTIVE_LIGAND_RECEPTOR_INTERACTION | -0.38 | -1.5 | 0.020 | 0.132 |
| KEGG_PROTEASOME | -0.54 | -1.49 | 0.130 | 0.132 |
| KEGG_SMALL_CELL_LUNG_CANCER | -0.41 | -1.49 | 0.056 | 0.134 |
| KEGG_GLIOMA | -0.4 | -1.48 | 0.052 | 0.137 |
| KEGG_HYPERTROPHIC_CARDIOMYOPATHY_HCM | -0.43 | -1.47 | 0.051 | 0.140 |
| KEGG_ETHER_LIPID_METABOLISM | -0.42 | -1.46 | 0.049 | 0.147 |
| KEGG_PROTEIN_EXPORT | -0.54 | -1.46 | 0.090 | 0.149 |
| KEGG_DORSO_VENTRAL_AXIS_FORMATION | -0.48 | -1.46 | 0.077 | 0.147 |
| KEGG_TRYPTOPHAN_METABOLISM | -0.44 | -1.45 | 0.055 | 0.150 |
| KEGG_GLYCOSAMINOGLYCAN_BIOSYNTHESIS_CHONDROITIN_SULFATE | -0.49 | -1.43 | 0.092 | 0.158 |
