# Supplementary material for: Necroptosis-related LncRNAs in skin cutaneous melanoma: evaluating prognosis, predicting immunity, and guiding therapy
Source: BMC Cancer. 2023 Aug 14;23:752. doi: 10.1186/s12885-023-11246-x (PMC10424397; doi:10.1186/s12885-023-11246-x)
Supplement: Supplementary file 4 — Supplementary Material 4 [file 12885_2023_11246_MOESM4_ESM.docx]

**Table S3. AL162457.2 Target Gene Prediction.**

| lncRNA | gene | geneType | cor | *P* value |
| --- | --- | --- | --- | --- |
| AL162457.2 | TIMM50 | protein_coding | 0.467 | 5.73E-27 |
| AL162457.2 | ATP2B1 | protein_coding | -0.637 | 5.40E-55 |
| AL162457.2 | GNG2 | protein_coding | -0.575 | 7.68E-43 |
| AL162457.2 | LAMA4 | protein_coding | -0.574 | 1.09E-42 |
| AL162457.2 | ARRDC3 | protein_coding | -0.567 | 1.65E-41 |
| AL162457.2 | STIM2 | protein_coding | -0.565 | 3.34E-41 |
| AL162457.2 | RIN2 | protein_coding | -0.564 | 5.56E-41 |
| AL162457.2 | ZEB1 | protein_coding | -0.552 | 6.48E-39 |
| AL162457.2 | AHR | protein_coding | -0.547 | 4.12E-38 |
| AL162457.2 | DOCK4 | protein_coding | -0.542 | 2.09E-37 |
| AL162457.2 | MMP16 | protein_coding | -0.542 | 2.23E-37 |
| AL162457.2 | BACH1 | protein_coding | -0.541 | 3.41E-37 |
| AL162457.2 | STARD13 | protein_coding | -0.541 | 3.53E-37 |
| AL162457.2 | MAML2 | protein_coding | -0.538 | 1.00E-36 |
| AL162457.2 | ITGA1 | protein_coding | -0.536 | 1.97E-36 |
| AL162457.2 | AGO3 | protein_coding | -0.534 | 3.52E-36 |
| AL162457.2 | CDK14 | protein_coding | -0.533 | 5.36E-36 |
| AL162457.2 | LMO4 | protein_coding | -0.531 | 1.16E-35 |
| AL162457.2 | CDK6 | protein_coding | -0.523 | 1.63E-34 |
| AL162457.2 | SNTB1 | protein_coding | -0.523 | 1.78E-34 |
| AL162457.2 | PDGFC | protein_coding | -0.521 | 3.85E-34 |
| AL162457.2 | PHACTR2 | protein_coding | -0.521 | 3.60E-34 |
| AL162457.2 | CFI | protein_coding | -0.514 | 3.47E-33 |
| AL162457.2 | TET3 | protein_coding | -0.511 | 8.50E-33 |
| AL162457.2 | DPYD | protein_coding | -0.509 | 1.81E-32 |
| AL162457.2 | ERRFI1 | protein_coding | -0.507 | 3.24E-32 |
| AL162457.2 | LOXL3 | protein_coding | -0.507 | 3.47E-32 |
| AL162457.2 | QKI | protein_coding | -0.503 | 1.43E-31 |
| AL162457.2 | ELK3 | protein_coding | -0.502 | 1.50E-31 |
| AL162457.2 | IL6ST | protein_coding | -0.501 | 2.53E-31 |
| AL162457.2 | CRIM1 | protein_coding | -0.5 | 3.14E-31 |
| AL162457.2 | RASA1 | protein_coding | -0.496 | 1.02E-30 |
| AL162457.2 | KCTD12 | protein_coding | -0.495 | 1.64E-30 |
| AL162457.2 | NRP1 | protein_coding | -0.495 | 1.74E-30 |
| AL162457.2 | AFAP1 | protein_coding | -0.49 | 6.47E-30 |
| AL162457.2 | RBMS3 | protein_coding | -0.49 | 6.41E-30 |
| AL162457.2 | SESN3 | protein_coding | -0.49 | 6.76E-30 |
| AL162457.2 | SLC5A3 | protein_coding | -0.489 | 8.51E-30 |
| AL162457.2 | TRPS1 | protein_coding | -0.488 | 1.16E-29 |
| AL162457.2 | GDNF | protein_coding | -0.487 | 1.90E-29 |
| AL162457.2 | NR3C1 | protein_coding | -0.486 | 2.16E-29 |
| AL162457.2 | SPOP | protein_coding | -0.485 | 3.40E-29 |
| AL162457.2 | FLT1 | protein_coding | -0.483 | 5.43E-29 |
| AL162457.2 | KHDRBS3 | protein_coding | -0.483 | 5.67E-29 |
| AL162457.2 | RFTN2 | protein_coding | -0.482 | 8.89E-29 |
| AL162457.2 | EPHB3 | protein_coding | -0.481 | 1.18E-28 |
| AL162457.2 | MAP4K4 | protein_coding | -0.481 | 1.16E-28 |
| AL162457.2 | RESF1 | protein_coding | -0.48 | 1.54E-28 |
| AL162457.2 | CEP85L | protein_coding | -0.479 | 2.08E-28 |
| AL162457.2 | PHF21A | protein_coding | -0.479 | 1.96E-28 |
| AL162457.2 | PPP1R12A | protein_coding | -0.479 | 1.70E-28 |
| AL162457.2 | MANEA | protein_coding | -0.478 | 2.55E-28 |
| AL162457.2 | DCP2 | protein_coding | -0.477 | 3.52E-28 |
| AL162457.2 | DIXDC1 | protein_coding | -0.476 | 5.31E-28 |
| AL162457.2 | PRKACB | protein_coding | -0.476 | 4.71E-28 |
| AL162457.2 | SSBP2 | protein_coding | -0.476 | 5.27E-28 |
| AL162457.2 | DLC1 | protein_coding | -0.475 | 6.64E-28 |
| AL162457.2 | GLIPR1 | protein_coding | -0.475 | 6.35E-28 |
| AL162457.2 | ZFHX3 | protein_coding | -0.472 | 1.33E-27 |
| AL162457.2 | DPYSL3 | protein_coding | -0.471 | 2.14E-27 |
| AL162457.2 | RAP1A | protein_coding | -0.471 | 1.74E-27 |
| AL162457.2 | ITM2B | protein_coding | -0.47 | 2.27E-27 |
| AL162457.2 | SEMA3A | protein_coding | -0.47 | 2.71E-27 |
| AL162457.2 | ITPRID2 | protein_coding | -0.468 | 4.94E-27 |
| AL162457.2 | NR2F2 | protein_coding | -0.468 | 4.40E-27 |
| AL162457.2 | SPRY4 | protein_coding | -0.468 | 4.62E-27 |
| AL162457.2 | ANGPT1 | protein_coding | -0.466 | 8.29E-27 |
| AL162457.2 | B3GNT5 | protein_coding | -0.465 | 9.57E-27 |
| AL162457.2 | C5orf15 | protein_coding | -0.465 | 1.20E-26 |
| AL162457.2 | CDK19 | protein_coding | -0.465 | 9.88E-27 |
| AL162457.2 | RBPJ | protein_coding | -0.465 | 1.14E-26 |
| AL162457.2 | TOP1 | protein_coding | -0.465 | 1.18E-26 |
| AL162457.2 | ADAMTS9 | protein_coding | -0.464 | 1.36E-26 |
| AL162457.2 | HIVEP3 | protein_coding | -0.464 | 1.57E-26 |
| AL162457.2 | SOCS5 | protein_coding | -0.464 | 1.24E-26 |
| AL162457.2 | NFATC3 | protein_coding | -0.463 | 1.68E-26 |
| AL162457.2 | CBX5 | protein_coding | -0.462 | 2.62E-26 |
| AL162457.2 | SMURF2 | protein_coding | -0.462 | 2.31E-26 |
| AL162457.2 | UHRF1BP1L | protein_coding | -0.462 | 2.41E-26 |
| AL162457.2 | WWTR1 | protein_coding | -0.462 | 2.67E-26 |
| AL162457.2 | ACVR2A | protein_coding | -0.461 | 3.38E-26 |
| AL162457.2 | TMEM47 | protein_coding | -0.461 | 3.21E-26 |
| AL162457.2 | DSE | protein_coding | -0.46 | 4.38E-26 |
| AL162457.2 | POGLUT3 | protein_coding | -0.46 | 3.84E-26 |
| AL162457.2 | TBCEL | protein_coding | -0.46 | 4.91E-26 |
| AL162457.2 | ZFP36L1 | protein_coding | -0.46 | 4.10E-26 |
| AL162457.2 | CPE | protein_coding | -0.459 | 5.44E-26 |
| AL162457.2 | PRKCI | protein_coding | -0.459 | 5.85E-26 |
| AL162457.2 | TLE4 | protein_coding | -0.459 | 5.47E-26 |
| AL162457.2 | UBXN4 | protein_coding | -0.459 | 5.07E-26 |
| AL162457.2 | FGF2 | protein_coding | -0.458 | 7.99E-26 |
| AL162457.2 | KDM7A | protein_coding | -0.458 | 7.56E-26 |
| AL162457.2 | PELI1 | protein_coding | -0.457 | 8.61E-26 |
| AL162457.2 | ROCK2 | protein_coding | -0.457 | 1.03E-25 |
| AL162457.2 | KLHL5 | protein_coding | -0.456 | 1.47E-25 |
| AL162457.2 | SP3 | protein_coding | -0.456 | 1.37E-25 |
| AL162457.2 | TMEM123 | protein_coding | -0.456 | 1.13E-25 |
| AL162457.2 | AFAP1L2 | protein_coding | -0.455 | 1.89E-25 |
| AL162457.2 | FRMD6 | protein_coding | -0.455 | 1.78E-25 |
| AL162457.2 | TANK | protein_coding | -0.455 | 1.67E-25 |
| AL162457.2 | TCF4 | protein_coding | -0.455 | 1.90E-25 |
| AL162457.2 | ARID4B | protein_coding | -0.454 | 2.47E-25 |
| AL162457.2 | MGP | protein_coding | -0.454 | 2.27E-25 |
| AL162457.2 | KIAA0040 | protein_coding | -0.453 | 2.88E-25 |
| AL162457.2 | VCAN | protein_coding | -0.453 | 3.04E-25 |
| AL162457.2 | CWC27 | protein_coding | -0.452 | 3.96E-25 |
| AL162457.2 | EPB41L2 | protein_coding | -0.452 | 4.22E-25 |
| AL162457.2 | PRKCA | protein_coding | -0.452 | 3.95E-25 |
| AL162457.2 | PSD3 | protein_coding | -0.452 | 3.36E-25 |
| AL162457.2 | AMOTL1 | protein_coding | -0.451 | 4.58E-25 |
| AL162457.2 | MEF2A | protein_coding | -0.451 | 5.71E-25 |
| AL162457.2 | SCAF11 | protein_coding | -0.451 | 5.44E-25 |
| AL162457.2 | MBD5 | protein_coding | -0.45 | 6.29E-25 |
| AL162457.2 | PDS5B | protein_coding | -0.45 | 6.55E-25 |
| AL162457.2 | BCLAF1 | protein_coding | -0.449 | 8.13E-25 |
| AL162457.2 | REST | protein_coding | -0.449 | 8.75E-25 |
| AL162457.2 | RPS6KA3 | protein_coding | -0.449 | 9.56E-25 |
| AL162457.2 | SERTAD2 | protein_coding | -0.449 | 7.67E-25 |
| AL162457.2 | SMAD5 | protein_coding | -0.449 | 9.33E-25 |
| AL162457.2 | TMEM178B | protein_coding | -0.449 | 9.19E-25 |
| AL162457.2 | USP47 | protein_coding | -0.449 | 9.61E-25 |
| AL162457.2 | CMTM6 | protein_coding | -0.448 | 1.15E-24 |
| AL162457.2 | FZD1 | protein_coding | -0.448 | 1.03E-24 |
| AL162457.2 | PALLD | protein_coding | -0.448 | 1.17E-24 |
| AL162457.2 | PHIP | protein_coding | -0.448 | 1.21E-24 |
| AL162457.2 | PPIL4 | protein_coding | -0.448 | 1.16E-24 |
| AL162457.2 | TMEM167A | protein_coding | -0.448 | 1.05E-24 |
| AL162457.2 | KALRN | protein_coding | -0.447 | 1.33E-24 |
| AL162457.2 | LIFR | protein_coding | -0.447 | 1.52E-24 |
| AL162457.2 | PLSCR4 | protein_coding | -0.447 | 1.38E-24 |
| AL162457.2 | CWC22 | protein_coding | -0.446 | 1.91E-24 |
| AL162457.2 | ABI2 | protein_coding | -0.445 | 2.44E-24 |
| AL162457.2 | YPEL2 | protein_coding | -0.445 | 2.36E-24 |
| AL162457.2 | EPC2 | protein_coding | -0.444 | 3.33E-24 |
| AL162457.2 | EPN2 | protein_coding | -0.444 | 3.62E-24 |
| AL162457.2 | MEF2C | protein_coding | -0.444 | 3.11E-24 |
| AL162457.2 | METTL7A | protein_coding | -0.444 | 3.42E-24 |
| AL162457.2 | ARHGEF6 | protein_coding | -0.443 | 4.27E-24 |
| AL162457.2 | BIRC2 | protein_coding | -0.443 | 4.13E-24 |
| AL162457.2 | BTBD3 | protein_coding | -0.443 | 3.74E-24 |
| AL162457.2 | ITGB3 | protein_coding | -0.443 | 4.49E-24 |
| AL162457.2 | SORL1 | protein_coding | -0.443 | 3.90E-24 |
| AL162457.2 | VEZT | protein_coding | -0.443 | 4.17E-24 |
| AL162457.2 | ZDHHC20 | protein_coding | -0.443 | 4.07E-24 |
| AL162457.2 | ATP11C | protein_coding | -0.442 | 5.28E-24 |
| AL162457.2 | NAB1 | protein_coding | -0.442 | 5.23E-24 |
| AL162457.2 | RNF138 | protein_coding | -0.442 | 5.81E-24 |
| AL162457.2 | TIA1 | protein_coding | -0.442 | 5.63E-24 |
| AL162457.2 | ITGA2 | protein_coding | -0.441 | 7.61E-24 |
| AL162457.2 | CNOT8 | protein_coding | -0.44 | 8.33E-24 |
| AL162457.2 | GABPA | protein_coding | -0.44 | 8.26E-24 |
| AL162457.2 | AGMO | protein_coding | -0.439 | 1.13E-23 |
| AL162457.2 | CLIC4 | protein_coding | -0.439 | 1.16E-23 |
| AL162457.2 | LPAR1 | protein_coding | -0.439 | 1.27E-23 |
| AL162457.2 | PRICKLE2 | protein_coding | -0.439 | 1.05E-23 |
| AL162457.2 | RARB | protein_coding | -0.439 | 1.30E-23 |
| AL162457.2 | SETD2 | protein_coding | -0.439 | 1.07E-23 |
| AL162457.2 | SNX1 | protein_coding | -0.439 | 1.33E-23 |
| AL162457.2 | TCF12 | protein_coding | -0.439 | 1.19E-23 |
| AL162457.2 | TMTC3 | protein_coding | -0.439 | 1.07E-23 |
| AL162457.2 | ATF2 | protein_coding | -0.438 | 1.51E-23 |
| AL162457.2 | CSGALNACT1 | protein_coding | -0.438 | 1.37E-23 |
| AL162457.2 | LMO7 | protein_coding | -0.438 | 1.60E-23 |
| AL162457.2 | PIBF1 | protein_coding | -0.438 | 1.41E-23 |
| AL162457.2 | ADAM17 | protein_coding | -0.437 | 1.76E-23 |
| AL162457.2 | ARL15 | protein_coding | -0.437 | 1.74E-23 |
| AL162457.2 | GOLIM4 | protein_coding | -0.437 | 2.18E-23 |
| AL162457.2 | ARSJ | protein_coding | -0.436 | 2.86E-23 |
| AL162457.2 | CREB1 | protein_coding | -0.436 | 2.30E-23 |
| AL162457.2 | MSANTD4 | protein_coding | -0.436 | 2.48E-23 |
| AL162457.2 | PLAGL1 | protein_coding | -0.436 | 2.68E-23 |
| AL162457.2 | SNRPB2 | protein_coding | -0.436 | 2.44E-23 |
| AL162457.2 | ATAD2B | protein_coding | -0.435 | 3.18E-23 |
| AL162457.2 | ATRX | protein_coding | -0.435 | 3.02E-23 |
| AL162457.2 | C16orf87 | protein_coding | -0.435 | 3.35E-23 |
| AL162457.2 | CASK | protein_coding | -0.435 | 3.54E-23 |
| AL162457.2 | N4BP2 | protein_coding | -0.435 | 3.34E-23 |
| AL162457.2 | NID1 | protein_coding | -0.435 | 3.52E-23 |
| AL162457.2 | PLOD2 | protein_coding | -0.435 | 3.41E-23 |
| AL162457.2 | RSPRY1 | protein_coding | -0.435 | 3.55E-23 |
| AL162457.2 | ITIH6 | protein_coding | -0.434 | 4.10E-23 |
| AL162457.2 | RND3 | protein_coding | -0.434 | 4.41E-23 |
| AL162457.2 | TENT5A | protein_coding | -0.434 | 4.33E-23 |
| AL162457.2 | TMEM30A | protein_coding | -0.434 | 4.58E-23 |
| AL162457.2 | VHL | protein_coding | -0.434 | 4.66E-23 |
| AL162457.2 | EMILIN1 | protein_coding | -0.433 | 5.36E-23 |
| AL162457.2 | GCNT1 | protein_coding | -0.433 | 5.15E-23 |
| AL162457.2 | MOSMO | protein_coding | -0.433 | 5.23E-23 |
| AL162457.2 | OSMR | protein_coding | -0.433 | 5.50E-23 |
| AL162457.2 | PLCE1 | protein_coding | -0.433 | 5.45E-23 |
| AL162457.2 | SLC39A10 | protein_coding | -0.433 | 5.80E-23 |
| AL162457.2 | ELF2 | protein_coding | -0.432 | 6.84E-23 |
| AL162457.2 | FEZ2 | protein_coding | -0.432 | 6.70E-23 |
| AL162457.2 | HSP90B1 | protein_coding | -0.432 | 6.70E-23 |
| AL162457.2 | PCM1 | protein_coding | -0.432 | 7.69E-23 |
| AL162457.2 | SLIT2 | protein_coding | -0.432 | 7.57E-23 |
| AL162457.2 | SMAD7 | protein_coding | -0.432 | 7.06E-23 |
| AL162457.2 | THAP2 | protein_coding | -0.432 | 6.84E-23 |
| AL162457.2 | UBTD2 | protein_coding | -0.432 | 6.24E-23 |
| AL162457.2 | FAM78B | protein_coding | -0.431 | 9.64E-23 |
| AL162457.2 | FLRT3 | protein_coding | -0.431 | 9.26E-23 |
| AL162457.2 | KLF9 | protein_coding | -0.431 | 9.51E-23 |
| AL162457.2 | B4GALT6 | protein_coding | -0.43 | 1.28E-22 |
| AL162457.2 | FREM1 | protein_coding | -0.43 | 1.21E-22 |
| AL162457.2 | FUT8 | protein_coding | -0.43 | 1.08E-22 |
| AL162457.2 | HELB | protein_coding | -0.43 | 1.26E-22 |
| AL162457.2 | MIS18BP1 | protein_coding | -0.43 | 1.03E-22 |
| AL162457.2 | NFAT5 | protein_coding | -0.43 | 1.08E-22 |
| AL162457.2 | NIPBL | protein_coding | -0.43 | 1.28E-22 |
| AL162457.2 | SMC5 | protein_coding | -0.43 | 1.06E-22 |
| AL162457.2 | STAMBPL1 | protein_coding | -0.43 | 1.15E-22 |
| AL162457.2 | ZMYM2 | protein_coding | -0.43 | 1.11E-22 |
| AL162457.2 | ATF7IP | protein_coding | -0.429 | 1.35E-22 |
| AL162457.2 | MED13L | protein_coding | -0.429 | 1.36E-22 |
| AL162457.2 | MTX3 | protein_coding | -0.429 | 1.57E-22 |
| AL162457.2 | PPIG | protein_coding | -0.429 | 1.62E-22 |
| AL162457.2 | TDG | protein_coding | -0.429 | 1.39E-22 |
| AL162457.2 | RAD21 | protein_coding | -0.428 | 2.00E-22 |
| AL162457.2 | RGS5 | protein_coding | -0.428 | 1.82E-22 |
| AL162457.2 | SELENOP | protein_coding | -0.428 | 1.73E-22 |
| AL162457.2 | FNDC3B | protein_coding | -0.427 | 2.55E-22 |
| AL162457.2 | RIF1 | protein_coding | -0.427 | 2.22E-22 |
| AL162457.2 | SLC4A7 | protein_coding | -0.427 | 2.73E-22 |
| AL162457.2 | TLR2 | protein_coding | -0.427 | 2.53E-22 |
| AL162457.2 | GOPC | protein_coding | -0.426 | 2.93E-22 |
| AL162457.2 | GTDC1 | protein_coding | -0.426 | 2.94E-22 |
| AL162457.2 | PPP2R2B | protein_coding | -0.426 | 2.91E-22 |
| AL162457.2 | SPAST | protein_coding | -0.426 | 3.13E-22 |
| AL162457.2 | ZNF292 | protein_coding | -0.426 | 3.51E-22 |
| AL162457.2 | ARFIP1 | protein_coding | -0.425 | 4.14E-22 |
| AL162457.2 | BBS10 | protein_coding | -0.425 | 4.06E-22 |
| AL162457.2 | CSNK1G3 | protein_coding | -0.425 | 4.05E-22 |
| AL162457.2 | GPC4 | protein_coding | -0.425 | 4.19E-22 |
| AL162457.2 | GPR34 | protein_coding | -0.425 | 4.43E-22 |
| AL162457.2 | PXYLP1 | protein_coding | -0.425 | 4.10E-22 |
| AL162457.2 | ZCCHC24 | protein_coding | -0.425 | 4.50E-22 |
| AL162457.2 | FAM172A | protein_coding | -0.424 | 4.71E-22 |
| AL162457.2 | PJA2 | protein_coding | -0.424 | 5.75E-22 |
| AL162457.2 | PTPN12 | protein_coding | -0.424 | 5.22E-22 |
| AL162457.2 | ROCK1 | protein_coding | -0.424 | 5.09E-22 |
| AL162457.2 | TRIM9 | protein_coding | -0.424 | 5.03E-22 |
| AL162457.2 | TXNDC15 | protein_coding | -0.424 | 5.46E-22 |
| AL162457.2 | FAM135A | protein_coding | -0.423 | 6.96E-22 |
| AL162457.2 | KCNK5 | protein_coding | -0.423 | 6.54E-22 |
| AL162457.2 | LAMB1 | protein_coding | -0.423 | 5.86E-22 |
| AL162457.2 | MBNL3 | protein_coding | -0.423 | 7.18E-22 |
| AL162457.2 | PHF10 | protein_coding | -0.423 | 6.10E-22 |
| AL162457.2 | RNF217 | protein_coding | -0.423 | 7.26E-22 |
| AL162457.2 | YES1 | protein_coding | -0.423 | 6.05E-22 |
| AL162457.2 | B3GALT1 | protein_coding | -0.422 | 8.57E-22 |
| AL162457.2 | FBXW7 | protein_coding | -0.422 | 8.05E-22 |
| AL162457.2 | IFT57 | protein_coding | -0.422 | 8.95E-22 |
| AL162457.2 | ITGAV | protein_coding | -0.422 | 7.87E-22 |
| AL162457.2 | PRRX1 | protein_coding | -0.422 | 7.66E-22 |
| AL162457.2 | RBMS1 | protein_coding | -0.422 | 8.28E-22 |
| AL162457.2 | STAG2 | protein_coding | -0.422 | 7.77E-22 |
| AL162457.2 | TOP2B | protein_coding | -0.422 | 8.25E-22 |
| AL162457.2 | ASF1A | protein_coding | -0.421 | 1.19E-21 |
| AL162457.2 | ERGIC2 | protein_coding | -0.421 | 9.94E-22 |
| AL162457.2 | PRPF38A | protein_coding | -0.421 | 1.08E-21 |
| AL162457.2 | PTPRZ1 | protein_coding | -0.421 | 1.15E-21 |
| AL162457.2 | ST6GAL1 | protein_coding | -0.421 | 1.09E-21 |
| AL162457.2 | ST8SIA4 | protein_coding | -0.421 | 1.10E-21 |
| AL162457.2 | USP34 | protein_coding | -0.421 | 1.06E-21 |
| AL162457.2 | ZNF146 | protein_coding | -0.421 | 1.08E-21 |
| AL162457.2 | MAP3K4 | protein_coding | -0.42 | 1.51E-21 |
| AL162457.2 | NCOA1 | protein_coding | -0.42 | 1.37E-21 |
| AL162457.2 | NFYB | protein_coding | -0.42 | 1.42E-21 |
| AL162457.2 | NR2F1 | protein_coding | -0.419 | 1.77E-21 |
| AL162457.2 | NREP | protein_coding | -0.419 | 1.84E-21 |
| AL162457.2 | PAPSS1 | protein_coding | -0.419 | 1.63E-21 |
| AL162457.2 | PTPRG | protein_coding | -0.419 | 1.79E-21 |
| AL162457.2 | S1PR2 | protein_coding | -0.419 | 1.92E-21 |
| AL162457.2 | SPRY2 | protein_coding | -0.419 | 1.84E-21 |
| AL162457.2 | AASDHPPT | protein_coding | -0.418 | 2.38E-21 |
| AL162457.2 | IGFBP5 | protein_coding | -0.418 | 2.39E-21 |
| AL162457.2 | LATS1 | protein_coding | -0.418 | 2.12E-21 |
| AL162457.2 | CEP120 | protein_coding | -0.417 | 2.73E-21 |
| AL162457.2 | FBXL3 | protein_coding | -0.417 | 2.77E-21 |
| AL162457.2 | HECA | protein_coding | -0.417 | 2.75E-21 |
| AL162457.2 | HOOK3 | protein_coding | -0.417 | 2.60E-21 |
| AL162457.2 | RCAN2 | protein_coding | -0.417 | 3.14E-21 |
| AL162457.2 | STRA6 | protein_coding | -0.417 | 2.81E-21 |
| AL162457.2 | TRIM5 | protein_coding | -0.417 | 2.77E-21 |
| AL162457.2 | ZDHHC17 | protein_coding | -0.417 | 2.77E-21 |
| AL162457.2 | ZNF184 | protein_coding | -0.417 | 2.82E-21 |
| AL162457.2 | AKAP6 | protein_coding | -0.416 | 3.91E-21 |
| AL162457.2 | DEPTOR | protein_coding | -0.416 | 3.24E-21 |
| AL162457.2 | DLG4 | protein_coding | -0.416 | 3.79E-21 |
| AL162457.2 | DNAJC10 | protein_coding | -0.416 | 3.44E-21 |
| AL162457.2 | EFCAB13 | protein_coding | -0.416 | 3.19E-21 |
| AL162457.2 | FCHO2 | protein_coding | -0.416 | 3.65E-21 |
| AL162457.2 | PHF14 | protein_coding | -0.416 | 3.83E-21 |
| AL162457.2 | ATF1 | protein_coding | -0.415 | 4.73E-21 |
| AL162457.2 | RHOU | protein_coding | -0.415 | 4.82E-21 |
| AL162457.2 | ZFP37 | protein_coding | -0.415 | 4.03E-21 |
| AL162457.2 | ZNF227 | protein_coding | -0.415 | 4.70E-21 |
| AL162457.2 | FAM3C | protein_coding | -0.414 | 5.87E-21 |
| AL162457.2 | HSPA13 | protein_coding | -0.414 | 6.06E-21 |
| AL162457.2 | LIMS1 | protein_coding | -0.414 | 5.14E-21 |
| AL162457.2 | ZFR | protein_coding | -0.414 | 6.01E-21 |
| AL162457.2 | CCDC102B | protein_coding | -0.413 | 7.47E-21 |
| AL162457.2 | CEP126 | protein_coding | -0.413 | 7.92E-21 |
| AL162457.2 | HECW2 | protein_coding | -0.413 | 6.44E-21 |
| AL162457.2 | IPO8 | protein_coding | -0.413 | 7.29E-21 |
| AL162457.2 | PGAP1 | protein_coding | -0.413 | 6.83E-21 |
| AL162457.2 | POGLUT1 | protein_coding | -0.413 | 7.19E-21 |
| AL162457.2 | RGL1 | protein_coding | -0.413 | 8.07E-21 |
| AL162457.2 | SEPTIN2 | protein_coding | -0.413 | 6.59E-21 |
| AL162457.2 | XIAP | protein_coding | -0.413 | 7.81E-21 |
| AL162457.2 | ZNF143 | protein_coding | -0.413 | 7.53E-21 |
| AL162457.2 | CFH | protein_coding | -0.412 | 9.79E-21 |
| AL162457.2 | HBEGF | protein_coding | -0.412 | 8.91E-21 |
| AL162457.2 | PDIA6 | protein_coding | -0.412 | 9.01E-21 |
| AL162457.2 | PPP1R9A | protein_coding | -0.412 | 8.54E-21 |
| AL162457.2 | ZNF260 | protein_coding | -0.412 | 9.02E-21 |
| AL162457.2 | ANTXR2 | protein_coding | -0.411 | 1.08E-20 |
| AL162457.2 | C11orf54 | protein_coding | -0.411 | 1.16E-20 |
| AL162457.2 | CMTM1 | protein_coding | -0.411 | 1.18E-20 |
| AL162457.2 | GPBP1 | protein_coding | -0.411 | 1.22E-20 |
| AL162457.2 | ITPRIPL2 | protein_coding | -0.411 | 1.12E-20 |
| AL162457.2 | KIAA0753 | protein_coding | -0.411 | 1.28E-20 |
| AL162457.2 | LAMC1 | protein_coding | -0.411 | 1.13E-20 |
| AL162457.2 | MAN1A2 | protein_coding | -0.411 | 1.23E-20 |
| AL162457.2 | P2RY1 | protein_coding | -0.411 | 1.09E-20 |
| AL162457.2 | PPP4R3B | protein_coding | -0.411 | 1.25E-20 |
| AL162457.2 | SDC2 | protein_coding | -0.411 | 1.12E-20 |
| AL162457.2 | SPATA6 | protein_coding | -0.411 | 1.25E-20 |
| AL162457.2 | TFPI | protein_coding | -0.411 | 1.29E-20 |
| AL162457.2 | ZNF41 | protein_coding | -0.411 | 1.13E-20 |
| AL162457.2 | FAM241A | protein_coding | -0.41 | 1.51E-20 |
| AL162457.2 | GOLPH3 | protein_coding | -0.41 | 1.59E-20 |
| AL162457.2 | HOXB3 | protein_coding | -0.41 | 1.33E-20 |
| AL162457.2 | MAP3K7 | protein_coding | -0.41 | 1.64E-20 |
| AL162457.2 | UBR3 | protein_coding | -0.41 | 1.63E-20 |
| AL162457.2 | GLG1 | protein_coding | -0.409 | 1.92E-20 |
| AL162457.2 | GPR155 | protein_coding | -0.409 | 1.75E-20 |
| AL162457.2 | KIAA0825 | protein_coding | -0.409 | 2.07E-20 |
| AL162457.2 | TENT2 | protein_coding | -0.409 | 1.66E-20 |
| AL162457.2 | ADGRL2 | protein_coding | -0.408 | 2.42E-20 |
| AL162457.2 | APPL2 | protein_coding | -0.408 | 2.13E-20 |
| AL162457.2 | LEMD3 | protein_coding | -0.408 | 2.25E-20 |
| AL162457.2 | MEOX2 | protein_coding | -0.408 | 2.39E-20 |
| AL162457.2 | NEK7 | protein_coding | -0.408 | 2.55E-20 |
| AL162457.2 | NUP160 | protein_coding | -0.408 | 2.19E-20 |
| AL162457.2 | RO60 | protein_coding | -0.408 | 2.28E-20 |
| AL162457.2 | ZNF639 | protein_coding | -0.408 | 2.38E-20 |
| AL162457.2 | FAM214A | protein_coding | -0.407 | 3.05E-20 |
| AL162457.2 | HS2ST1 | protein_coding | -0.407 | 2.97E-20 |
| AL162457.2 | JMJD1C | protein_coding | -0.407 | 2.81E-20 |
| AL162457.2 | KANSL1L | protein_coding | -0.407 | 3.01E-20 |
| AL162457.2 | KPNA5 | protein_coding | -0.407 | 2.68E-20 |
| AL162457.2 | NFIA | protein_coding | -0.407 | 3.30E-20 |
| AL162457.2 | RBM7 | protein_coding | -0.407 | 3.02E-20 |
| AL162457.2 | SERINC3 | protein_coding | -0.407 | 2.69E-20 |
| AL162457.2 | SETD7 | protein_coding | -0.407 | 3.26E-20 |
| AL162457.2 | TAB2 | protein_coding | -0.407 | 3.16E-20 |
| AL162457.2 | UTP23 | protein_coding | -0.407 | 2.65E-20 |
| AL162457.2 | ZNF136 | protein_coding | -0.407 | 3.29E-20 |
| AL162457.2 | BMP6 | protein_coding | -0.406 | 3.36E-20 |
| AL162457.2 | CWF19L2 | protein_coding | -0.406 | 3.97E-20 |
| AL162457.2 | KRR1 | protein_coding | -0.406 | 3.80E-20 |
| AL162457.2 | MOB1A | protein_coding | -0.406 | 3.75E-20 |
| AL162457.2 | NFE2L3 | protein_coding | -0.406 | 3.44E-20 |
| AL162457.2 | NUDT21 | protein_coding | -0.406 | 4.02E-20 |
| AL162457.2 | SKIL | protein_coding | -0.406 | 3.74E-20 |
| AL162457.2 | ARID4A | protein_coding | -0.405 | 4.51E-20 |
| AL162457.2 | DLX1 | protein_coding | -0.405 | 4.73E-20 |
| AL162457.2 | ERLEC1 | protein_coding | -0.405 | 5.03E-20 |
| AL162457.2 | FANCL | protein_coding | -0.405 | 4.35E-20 |
| AL162457.2 | FREM2 | protein_coding | -0.405 | 5.12E-20 |
| AL162457.2 | GOLM2 | protein_coding | -0.405 | 4.59E-20 |
| AL162457.2 | PGRMC1 | protein_coding | -0.405 | 4.88E-20 |
| AL162457.2 | PPFIBP1 | protein_coding | -0.405 | 4.94E-20 |
| AL162457.2 | ZRANB2 | protein_coding | -0.405 | 4.31E-20 |
| AL162457.2 | C1QTNF3 | protein_coding | -0.404 | 5.62E-20 |
| AL162457.2 | CCN2 | protein_coding | -0.404 | 6.04E-20 |
| AL162457.2 | CD302 | protein_coding | -0.404 | 6.01E-20 |
| AL162457.2 | CDH2 | protein_coding | -0.404 | 6.51E-20 |
| AL162457.2 | CHN1 | protein_coding | -0.404 | 6.07E-20 |
| AL162457.2 | COL8A1 | protein_coding | -0.404 | 5.51E-20 |
| AL162457.2 | CPD | protein_coding | -0.404 | 5.67E-20 |
| AL162457.2 | PDGFA | protein_coding | -0.404 | 6.47E-20 |
| AL162457.2 | XRCC4 | protein_coding | -0.404 | 6.51E-20 |
| AL162457.2 | CTDSPL2 | protein_coding | -0.403 | 8.14E-20 |
| AL162457.2 | DENND4A | protein_coding | -0.403 | 8.15E-20 |
| AL162457.2 | F13A1 | protein_coding | -0.403 | 7.38E-20 |
| AL162457.2 | KLHL20 | protein_coding | -0.403 | 7.07E-20 |
| AL162457.2 | PAFAH1B2 | protein_coding | -0.403 | 7.22E-20 |
| AL162457.2 | ZNF638 | protein_coding | -0.403 | 6.90E-20 |
| AL162457.2 | ZNF660 | protein_coding | -0.403 | 7.04E-20 |
| AL162457.2 | CHIC2 | protein_coding | -0.402 | 9.32E-20 |
| AL162457.2 | DPF3 | protein_coding | -0.402 | 8.32E-20 |
| AL162457.2 | HOXA3 | protein_coding | -0.402 | 8.94E-20 |
| AL162457.2 | IBTK | protein_coding | -0.402 | 8.55E-20 |
| AL162457.2 | IGFBP3 | protein_coding | -0.402 | 9.35E-20 |
| AL162457.2 | ITGA6 | protein_coding | -0.402 | 8.94E-20 |
| AL162457.2 | JMY | protein_coding | -0.402 | 9.08E-20 |
| AL162457.2 | PAPOLG | protein_coding | -0.402 | 9.52E-20 |
| AL162457.2 | SEC24D | protein_coding | -0.402 | 1.03E-19 |
| AL162457.2 | SGMS1 | protein_coding | -0.402 | 9.01E-20 |
| AL162457.2 | TRIL | protein_coding | -0.402 | 9.83E-20 |
| AL162457.2 | AFTPH | protein_coding | -0.401 | 1.17E-19 |
| AL162457.2 | CUL4B | protein_coding | -0.401 | 1.27E-19 |
| AL162457.2 | MTMR6 | protein_coding | -0.401 | 1.11E-19 |
| AL162457.2 | PGM2 | protein_coding | -0.401 | 1.18E-19 |
| AL162457.2 | PLCB1 | protein_coding | -0.401 | 1.30E-19 |
| AL162457.2 | RANBP2 | protein_coding | -0.401 | 1.16E-19 |
| AL162457.2 | TJP1 | protein_coding | -0.401 | 1.13E-19 |
| AL162457.2 | TMTC2 | protein_coding | -0.401 | 1.24E-19 |
| AL162457.2 | ANKH | protein_coding | -0.4 | 1.46E-19 |
| AL162457.2 | CCP110 | protein_coding | -0.4 | 1.54E-19 |
| AL162457.2 | IGFBP2 | protein_coding | -0.4 | 1.62E-19 |
| AL162457.2 | ITGA4 | protein_coding | -0.4 | 1.37E-19 |
| AL162457.2 | NEGR1 | protein_coding | -0.4 | 1.57E-19 |
| AL162457.2 | REV1 | protein_coding | -0.4 | 1.43E-19 |
| AL162457.2 | TMX3 | protein_coding | -0.4 | 1.53E-19 |
| AL162457.2 | CD63 | protein_coding | 0.4 | 1.53E-19 |
| AL162457.2 | ERFE | protein_coding | 0.4 | 1.51E-19 |
| AL162457.2 | ORAI3 | protein_coding | 0.4 | 1.42E-19 |
| AL162457.2 | UQCRC1 | protein_coding | 0.401 | 1.29E-19 |
| AL162457.2 | BIRC7 | protein_coding | 0.402 | 9.04E-20 |
| AL162457.2 | GPRC5A | protein_coding | 0.402 | 1.01E-19 |
| AL162457.2 | HEBP1 | protein_coding | 0.402 | 8.40E-20 |
| AL162457.2 | RAB17 | protein_coding | 0.402 | 9.97E-20 |
| AL162457.2 | TPPP | protein_coding | 0.402 | 8.50E-20 |
| AL162457.2 | ZDHHC16 | protein_coding | 0.402 | 8.29E-20 |
| AL162457.2 | HS1BP3 | protein_coding | 0.403 | 6.68E-20 |
| AL162457.2 | IRX3 | protein_coding | 0.403 | 6.79E-20 |
| AL162457.2 | UMODL1 | protein_coding | 0.403 | 7.86E-20 |
| AL162457.2 | HMG20B | protein_coding | 0.404 | 5.34E-20 |
| AL162457.2 | MITF | protein_coding | 0.404 | 5.61E-20 |
| AL162457.2 | PITRM1 | protein_coding | 0.406 | 3.72E-20 |
| AL162457.2 | RRP1 | protein_coding | 0.407 | 3.18E-20 |
| AL162457.2 | SLC66A1 | protein_coding | 0.407 | 2.87E-20 |
| AL162457.2 | VAC14 | protein_coding | 0.407 | 3.18E-20 |
| AL162457.2 | ATP6AP1 | protein_coding | 0.408 | 2.35E-20 |
| AL162457.2 | HCFC1R1 | protein_coding | 0.408 | 2.44E-20 |
| AL162457.2 | RAB5B | protein_coding | 0.408 | 2.25E-20 |
| AL162457.2 | SIRPA | protein_coding | 0.408 | 2.46E-20 |
| AL162457.2 | TUBB4A | protein_coding | 0.408 | 2.31E-20 |
| AL162457.2 | UROS | protein_coding | 0.408 | 2.11E-20 |
| AL162457.2 | BAMBI | protein_coding | 0.409 | 1.97E-20 |
| AL162457.2 | S100A11 | protein_coding | 0.409 | 1.65E-20 |
| AL162457.2 | TYR | protein_coding | 0.409 | 1.84E-20 |
| AL162457.2 | TMEM251 | protein_coding | 0.41 | 1.51E-20 |
| AL162457.2 | ITGB1BP1 | protein_coding | 0.411 | 1.24E-20 |
| AL162457.2 | NECAB2 | protein_coding | 0.411 | 1.07E-20 |
| AL162457.2 | SLC5A10 | protein_coding | 0.411 | 1.30E-20 |
| AL162457.2 | SMIM4 | protein_coding | 0.411 | 1.07E-20 |
| AL162457.2 | TINCR | protein_coding | 0.411 | 1.17E-20 |
| AL162457.2 | VEGFB | protein_coding | 0.411 | 1.15E-20 |
| AL162457.2 | CHCHD6 | protein_coding | 0.414 | 6.07E-21 |
| AL162457.2 | SEMA6A | protein_coding | 0.414 | 5.14E-21 |
| AL162457.2 | TSPO | protein_coding | 0.414 | 5.37E-21 |
| AL162457.2 | GALK1 | protein_coding | 0.415 | 4.31E-21 |
| AL162457.2 | IPO13 | protein_coding | 0.415 | 4.62E-21 |
| AL162457.2 | OSGIN1 | protein_coding | 0.415 | 4.60E-21 |
| AL162457.2 | PEPD | protein_coding | 0.415 | 5.02E-21 |
| AL162457.2 | PRDM7 | protein_coding | 0.415 | 4.04E-21 |
| AL162457.2 | NCS1 | protein_coding | 0.416 | 3.62E-21 |
| AL162457.2 | SSU72 | protein_coding | 0.417 | 2.65E-21 |
| AL162457.2 | BACE2 | protein_coding | 0.418 | 2.33E-21 |
| AL162457.2 | MMP24OS | protein_coding | 0.418 | 2.03E-21 |
| AL162457.2 | NUDT8 | protein_coding | 0.418 | 2.06E-21 |
| AL162457.2 | PCYT2 | protein_coding | 0.418 | 1.98E-21 |
| AL162457.2 | RIPK4 | protein_coding | 0.418 | 2.33E-21 |
| AL162457.2 | TMEM138 | protein_coding | 0.418 | 2.13E-21 |
| AL162457.2 | RAB3A | protein_coding | 0.419 | 1.82E-21 |
| AL162457.2 | ATP6V0C | protein_coding | 0.42 | 1.44E-21 |
| AL162457.2 | ADGRG1 | protein_coding | 0.421 | 1.12E-21 |
| AL162457.2 | LGALS3 | protein_coding | 0.421 | 9.86E-22 |
| AL162457.2 | ADAM11 | protein_coding | 0.423 | 7.10E-22 |
| AL162457.2 | DIPK1C | protein_coding | 0.423 | 6.18E-22 |
| AL162457.2 | GMPR | protein_coding | 0.423 | 5.80E-22 |
| AL162457.2 | HPS6 | protein_coding | 0.424 | 5.50E-22 |
| AL162457.2 | TKT | protein_coding | 0.424 | 5.02E-22 |
| AL162457.2 | TOLLIP | protein_coding | 0.424 | 4.81E-22 |
| AL162457.2 | C11orf96 | protein_coding | 0.425 | 3.85E-22 |
| AL162457.2 | TFF3 | protein_coding | 0.426 | 3.46E-22 |
| AL162457.2 | UPP1 | protein_coding | 0.426 | 3.40E-22 |
| AL162457.2 | ATP6V0D2 | protein_coding | 0.429 | 1.38E-22 |
| AL162457.2 | MC1R | protein_coding | 0.429 | 1.34E-22 |
| AL162457.2 | SPACA3 | protein_coding | 0.429 | 1.35E-22 |
| AL162457.2 | AHNAK2 | protein_coding | 0.43 | 1.07E-22 |
| AL162457.2 | GLA | protein_coding | 0.431 | 9.67E-23 |
| AL162457.2 | LSS | protein_coding | 0.431 | 8.16E-23 |
| AL162457.2 | NAT8 | protein_coding | 0.431 | 8.80E-23 |
| AL162457.2 | CINP | protein_coding | 0.432 | 6.71E-23 |
| AL162457.2 | TFAP2A | protein_coding | 0.432 | 6.32E-23 |
| AL162457.2 | TNFRSF14 | protein_coding | 0.432 | 6.91E-23 |
| AL162457.2 | CFAP61 | protein_coding | 0.433 | 6.15E-23 |
| AL162457.2 | DHDH | protein_coding | 0.433 | 5.63E-23 |
| AL162457.2 | MBP | protein_coding | 0.433 | 4.86E-23 |
| AL162457.2 | TPBGL | protein_coding | 0.433 | 4.87E-23 |
| AL162457.2 | DPP7 | protein_coding | 0.434 | 4.04E-23 |
| AL162457.2 | GBA | protein_coding | 0.434 | 4.13E-23 |
| AL162457.2 | NDUFB4 | protein_coding | 0.434 | 4.67E-23 |
| AL162457.2 | STARD10 | protein_coding | 0.434 | 3.74E-23 |
| AL162457.2 | SLC38A8 | protein_coding | 0.435 | 3.17E-23 |
| AL162457.2 | TMEM268 | protein_coding | 0.435 | 3.30E-23 |
| AL162457.2 | USF2 | protein_coding | 0.435 | 3.16E-23 |
| AL162457.2 | RPP25 | protein_coding | 0.436 | 2.26E-23 |
| AL162457.2 | ST7 | protein_coding | 0.436 | 2.50E-23 |
| AL162457.2 | DYNLRB1 | protein_coding | 0.437 | 1.75E-23 |
| AL162457.2 | HMOX2 | protein_coding | 0.438 | 1.63E-23 |
| AL162457.2 | ARHGAP8 | protein_coding | 0.439 | 1.03E-23 |
| AL162457.2 | CELSR1 | protein_coding | 0.439 | 1.10E-23 |
| AL162457.2 | LHB | protein_coding | 0.44 | 1.01E-23 |
| AL162457.2 | MLANA | protein_coding | 0.441 | 7.01E-24 |
| AL162457.2 | NMRK2 | protein_coding | 0.441 | 6.65E-24 |
| AL162457.2 | NANS | protein_coding | 0.443 | 4.00E-24 |
| AL162457.2 | UBAP1L | protein_coding | 0.444 | 3.59E-24 |
| AL162457.2 | DEXI | protein_coding | 0.445 | 2.49E-24 |
| AL162457.2 | WHRN | protein_coding | 0.445 | 2.27E-24 |
| AL162457.2 | COMMD4 | protein_coding | 0.446 | 1.93E-24 |
| AL162457.2 | CYB561A3 | protein_coding | 0.446 | 1.70E-24 |
| AL162457.2 | TRPV2 | protein_coding | 0.447 | 1.45E-24 |
| AL162457.2 | FN3K | protein_coding | 0.448 | 1.06E-24 |
| AL162457.2 | MED15 | protein_coding | 0.448 | 9.89E-25 |
| AL162457.2 | PAK4 | protein_coding | 0.449 | 9.15E-25 |
| AL162457.2 | MIEN1 | protein_coding | 0.45 | 7.26E-25 |
| AL162457.2 | TYSND1 | protein_coding | 0.45 | 5.93E-25 |
| AL162457.2 | MRPS2 | protein_coding | 0.451 | 5.30E-25 |
| AL162457.2 | ANKRD39 | protein_coding | 0.452 | 3.67E-25 |
| AL162457.2 | CA14 | protein_coding | 0.452 | 4.02E-25 |
| AL162457.2 | ILVBL | protein_coding | 0.452 | 3.85E-25 |
| AL162457.2 | GYPC | protein_coding | 0.453 | 3.19E-25 |
| AL162457.2 | GLMP | protein_coding | 0.454 | 2.03E-25 |
| AL162457.2 | RABGGTA | protein_coding | 0.455 | 1.57E-25 |
| AL162457.2 | CLN6 | protein_coding | 0.457 | 1.07E-25 |
| AL162457.2 | PPM1H | protein_coding | 0.457 | 9.86E-26 |
| AL162457.2 | SLC25A39 | protein_coding | 0.459 | 5.63E-26 |
| AL162457.2 | AP5B1 | protein_coding | 0.46 | 3.99E-26 |
| AL162457.2 | ATOX1 | protein_coding | 0.46 | 4.16E-26 |
| AL162457.2 | CABLES1 | protein_coding | 0.461 | 3.68E-26 |
| AL162457.2 | MRPL41 | protein_coding | 0.461 | 3.44E-26 |
| AL162457.2 | ARFGAP1 | protein_coding | 0.465 | 1.18E-26 |
| AL162457.2 | SLC6A8 | protein_coding | 0.465 | 1.15E-26 |
| AL162457.2 | CNPPD1 | protein_coding | 0.466 | 8.60E-27 |
| AL162457.2 | LAGE3 | protein_coding | 0.466 | 8.65E-27 |
| AL162457.2 | RETSAT | protein_coding | 0.466 | 7.91E-27 |
| AL162457.2 | POU3F1 | protein_coding | 0.467 | 6.36E-27 |
| AL162457.2 | GSTP1 | protein_coding | 0.468 | 4.87E-27 |
| AL162457.2 | MYO1D | protein_coding | 0.469 | 3.67E-27 |
| AL162457.2 | SMPD2 | protein_coding | 0.469 | 3.21E-27 |
| AL162457.2 | IGSF8 | protein_coding | 0.47 | 2.45E-27 |
| AL162457.2 | SLC1A4 | protein_coding | 0.471 | 2.05E-27 |
| AL162457.2 | SLC27A3 | protein_coding | 0.471 | 2.24E-27 |
| AL162457.2 | PRICKLE3 | protein_coding | 0.475 | 5.93E-28 |
| AL162457.2 | TPRN | protein_coding | 0.475 | 5.73E-28 |
| AL162457.2 | PNMA6A | protein_coding | 0.477 | 3.37E-28 |
| AL162457.2 | UCK1 | protein_coding | 0.477 | 3.17E-28 |
| AL162457.2 | ZNF703 | protein_coding | 0.477 | 3.40E-28 |
| AL162457.2 | COMTD1 | protein_coding | 0.478 | 2.71E-28 |
| AL162457.2 | NR4A3 | protein_coding | 0.478 | 2.53E-28 |
| AL162457.2 | MGST3 | protein_coding | 0.479 | 1.68E-28 |
| AL162457.2 | TPCN2 | protein_coding | 0.479 | 1.73E-28 |
| AL162457.2 | SAMM50 | protein_coding | 0.48 | 1.44E-28 |
| AL162457.2 | SLC6A17 | protein_coding | 0.48 | 1.49E-28 |
| AL162457.2 | VEPH1 | protein_coding | 0.48 | 1.29E-28 |
| AL162457.2 | KCNIP3 | protein_coding | 0.481 | 1.08E-28 |
| AL162457.2 | SLC7A4 | protein_coding | 0.482 | 7.21E-29 |
| AL162457.2 | TOM1 | protein_coding | 0.483 | 6.37E-29 |
| AL162457.2 | VPS18 | protein_coding | 0.485 | 3.28E-29 |
| AL162457.2 | KREMEN2 | protein_coding | 0.488 | 1.16E-29 |
| AL162457.2 | RENBP | protein_coding | 0.49 | 7.41E-30 |
| AL162457.2 | AGPAT2 | protein_coding | 0.491 | 4.98E-30 |
| AL162457.2 | AMDHD2 | protein_coding | 0.491 | 5.81E-30 |
| AL162457.2 | FNDC10 | protein_coding | 0.492 | 4.08E-30 |
| AL162457.2 | SNTA1 | protein_coding | 0.493 | 2.58E-30 |
| AL162457.2 | H2AJ | protein_coding | 0.494 | 1.92E-30 |
| AL162457.2 | QPCT | protein_coding | 0.495 | 1.64E-30 |
| AL162457.2 | VAT1 | protein_coding | 0.496 | 1.11E-30 |
| AL162457.2 | DIPK1B | protein_coding | 0.497 | 7.08E-31 |
| AL162457.2 | NEDD4L | protein_coding | 0.497 | 8.84E-31 |
| AL162457.2 | GPR137B | protein_coding | 0.501 | 2.32E-31 |
| AL162457.2 | TANGO2 | protein_coding | 0.513 | 5.37E-33 |
| AL162457.2 | SNX8 | protein_coding | 0.514 | 3.64E-33 |
| AL162457.2 | BAIAP2L1 | protein_coding | 0.515 | 2.72E-33 |
| AL162457.2 | FLYWCH1 | protein_coding | 0.515 | 2.68E-33 |
| AL162457.2 | PSEN2 | protein_coding | 0.515 | 2.35E-33 |
| AL162457.2 | PIK3CD | protein_coding | 0.517 | 1.28E-33 |
| AL162457.2 | PPP1R37 | protein_coding | 0.518 | 8.06E-34 |
| AL162457.2 | MAD1L1 | protein_coding | 0.519 | 7.62E-34 |
| AL162457.2 | CLCN7 | protein_coding | 0.524 | 1.39E-34 |
| AL162457.2 | NAXE | protein_coding | 0.525 | 1.01E-34 |
| AL162457.2 | RAB32 | protein_coding | 0.527 | 5.09E-35 |
| AL162457.2 | SLC3A2 | protein_coding | 0.527 | 4.48E-35 |
| AL162457.2 | HAGHL | protein_coding | 0.53 | 1.35E-35 |
| AL162457.2 | EPHX1 | protein_coding | 0.534 | 4.43E-36 |
| AL162457.2 | QPCTL | protein_coding | 0.534 | 4.15E-36 |
| AL162457.2 | SLC45A2 | protein_coding | 0.534 | 3.79E-36 |
| AL162457.2 | TBC1D16 | protein_coding | 0.534 | 3.55E-36 |
| AL162457.2 | ATP6V1F | protein_coding | 0.535 | 2.80E-36 |
| AL162457.2 | MICAL1 | protein_coding | 0.535 | 2.99E-36 |
| AL162457.2 | SLC7A5 | protein_coding | 0.539 | 6.29E-37 |
| AL162457.2 | PAEP | protein_coding | 0.54 | 3.99E-37 |
| AL162457.2 | ABCD1 | protein_coding | 0.541 | 3.14E-37 |
| AL162457.2 | MLPH | protein_coding | 0.541 | 3.32E-37 |
| AL162457.2 | SCARB1 | protein_coding | 0.542 | 1.96E-37 |
| AL162457.2 | BRI3 | protein_coding | 0.547 | 4.12E-38 |
| AL162457.2 | RTN4R | protein_coding | 0.553 | 3.24E-39 |
| AL162457.2 | GOLGA7B | protein_coding | 0.555 | 1.99E-39 |
| AL162457.2 | MFSD12 | protein_coding | 0.555 | 1.78E-39 |
| AL162457.2 | CSTB | protein_coding | 0.565 | 3.94E-41 |
| AL162457.2 | GPR143 | protein_coding | 0.565 | 3.37E-41 |
| AL162457.2 | MGAT5B | protein_coding | 0.565 | 3.86E-41 |
| AL162457.2 | AVPI1 | protein_coding | 0.568 | 9.68E-42 |
| AL162457.2 | WIPI1 | protein_coding | 0.571 | 4.05E-42 |
| AL162457.2 | CDK2 | protein_coding | 0.574 | 9.45E-43 |
| AL162457.2 | SLC16A6 | protein_coding | 0.582 | 3.36E-44 |
| AL162457.2 | GSTO1 | protein_coding | 0.583 | 3.10E-44 |
| AL162457.2 | ANKRD9 | protein_coding | 0.589 | 1.86E-45 |
| AL162457.2 | ARSG | protein_coding | 0.589 | 1.68E-45 |
| AL162457.2 | HES6 | protein_coding | 0.59 | 1.13E-45 |
| AL162457.2 | TTYH2 | protein_coding | 0.591 | 7.38E-46 |
| AL162457.2 | ITPKB | protein_coding | 0.594 | 1.94E-46 |
| AL162457.2 | TRIM63 | protein_coding | 0.595 | 1.47E-46 |
| AL162457.2 | OCA2 | protein_coding | 0.613 | 5.73E-50 |
| AL162457.2 | PMEL | protein_coding | 0.618 | 4.15E-51 |
| AL162457.2 | KCNAB2 | protein_coding | 0.633 | 3.76E-54 |
| AL162457.2 | TRPM1 | protein_coding | 0.633 | 2.76E-54 |
| AL162457.2 | TSPAN10 | protein_coding | 0.638 | 2.94E-55 |
